# Comparative genomics of the dairy isolate *Streptococcus macedonicus* ACA-DC 198 against related members of the *Streptococcus bovis*/*Streptococcus equinus* complex

**DOI:** 10.1186/1471-2164-15-272

**Published:** 2014-04-08

**Authors:** Konstantinos Papadimitriou, Rania Anastasiou, Eleni Mavrogonatou, Jochen Blom, Nikos C Papandreou, Stavros J Hamodrakas, Stéphanie Ferreira, Pierre Renault, Philip Supply, Bruno Pot, Effie Tsakalidou

**Affiliations:** 1 Laboratory of Dairy Research, Department of Food Science and Human Nutrition, Agricultural University of Athens, Iera Odos 75, Athens 118 55, Greece; 2 Laboratory of Cell Proliferation and Ageing, Institute of Biosciences and Applications, National Centre for Scientific Research "Demokritos", Athens 153 10, Greece; 3 Computational Genomics, Center for Biotechnology, Bielefeld University, Bielefeld, Germany; 4 Department of Cell Biology and Biophysics, Faculty of Biology, University of Athens, Panepistimiopolis, Athens 157 01, Greece; 5 Genoscreen, Genomic Platform and R&D, Campus de l’Institut Pasteur, 1 rue du Professeur Calmette, Lille 59000, France; 6 INRA, UMR1319 Micalis, Jouy-en-Josas F-78352, France; 7 AgroParisTech, UMR Micalis, Jouy-en-Josas F-78352, France; 8 Institut Pasteur de Lille, Center for Infection and Immunity of Lille (CIIL), Lille F-59019, France; 9 Inserm U1019, F-59019 Lille, France; 10 CNRS UMR8204, Lille F-59021, France; 11 Univ Lille de Nord France, Lille F-59000, France

**Keywords:** *Streptococcus*, Genome, Adaptation, Gene decay, Pseudogene, Horizontal gene transfer, Pathogenicity, Virulence factor, Milk, Niche

## Abstract

**Background:**

Within the genus *Streptococcus*, only *Streptococcus thermophilus* is used as a starter culture in food fermentations. *Streptococcus macedonicus* though, which belongs to the *Streptococcus bovis*/*Streptococcus equinus* complex (SBSEC), is also frequently isolated from fermented foods mainly of dairy origin. Members of the SBSEC have been implicated in human endocarditis and colon cancer. Here we compare the genome sequence of the dairy isolate *S. macedonicus* ACA-DC 198 to the other SBSEC genomes in order to assess *in silico* its potential adaptation to milk and its pathogenicity status.

**Results:**

Despite the fact that the SBSEC species were found tightly related based on whole genome phylogeny of streptococci, two distinct patterns of evolution were identified among them. *Streptococcus macedonicus, Streptococcus infantarius* CJ18 and *Streptococcus pasteurianus* ATCC 43144 seem to have undergone reductive evolution resulting in significantly diminished genome sizes and increased percentages of potential pseudogenes when compared to *Streptococcus gallolyticus* subsp. *gallolyticus*. In addition, the three species seem to have lost genes for catabolizing complex plant carbohydrates and for detoxifying toxic substances previously linked to the ability of *S. gallolyticus* to survive in the rumen. Analysis of the *S. macedonicus* genome revealed features that could support adaptation to milk, including an extra gene cluster for lactose and galactose metabolism, a proteolytic system for casein hydrolysis, auxotrophy for several vitamins, an increased ability to resist bacteriophages and horizontal gene transfer events with the dairy *Lactococcus lactis* and *S. thermophilus* as potential donors. In addition, *S. macedonicus* lacks several pathogenicity-related genes found in *S. gallolyticus*. For example, *S. macedonicus* has retained only one (i.e. the *pil3*) of the three pilus gene clusters which may mediate the binding of *S. gallolyticus* to the extracellular matrix. Unexpectedly, similar findings were obtained not only for the dairy *S. infantarius* CJ18, but also for the blood isolate *S. pasteurianus* ATCC 43144.

**Conclusions:**

Our whole genome analyses suggest traits of adaptation of *S. macedonicus* to the nutrient-rich dairy environment. During this process the bacterium gained genes presumably important for this new ecological niche. Finally, *S. macedonicus* carries a reduced number of putative SBSEC virulence factors, which suggests a diminished pathogenic potential.

## Background

Lactic acid bacteria (LAB) constitute a very important group of microorganisms for the food industry, as well as the health of humans and animals [1,2]. Several species in this group have a long history of safe use in fermented foods and thus belong to the very few bacteria that may qualify for the "generally regarded as safe" (GRAS) or the "qualified presumption of safety" (QPS) status according to FDA and EFSA, respectively [3]. Other LAB species are commensals of the skin, the oral cavity, the respiratory system, the gastrointestinal tract (GIT) and the genitals of mammals or other organisms. Furthermore, the presence of specific LAB strains, called "probiotics", in certain niches of the body is considered to promote the health of the host [2]. This benign nature of LAB, as well as their economic value, often obscure the existence of notorious LAB pathogens that are among the leading causes of human morbidity and mortality worldwide [4].

This oxymoron about the vast differences in the pathogenic potential within the LAB group is probably best exemplified by streptococci. The genus basically consists of commensals that include several severe pathogens, like group A streptococci (GAS), group B streptococci (GBS) and *Streptococcus pneumoniae*[5]. Streptococcal pathogens are implicated in a plethora of diseases, ranging from mild (e.g. pharyngitis) to invasive and life-threatening (e.g. necrotizing fasciitis) infections [6]. In contrast, *Streptococcus thermophilus* is one of the most frequent starter LAB consumed by humans in yogurt and cheese [7]. It is believed that this is the only streptococcal species that, during its adaptation to the nutrient-rich milk environment, underwent extensive genome decay, resulting in the loss of pathogenicity-related genes present in members of the genus [7,8].

Apart from *S. thermophilus*, other streptococci can grow in milk and milk products. Such streptococci mainly belong to the *Streptococcus bovis*/*Streptococcus equinus* complex (SBSEC) [9]. The exact route that would explain their presence in milk is yet unidentified. In theory, since some of them can naturally occur in the GIT or on the teat skin of lactating animals, they could be passively transmitted to raw milk. In addition, species of the SBSEC are known to be involved in human cases of endocarditis, meningitis, bacteremia and colon cancer [10-12]. However, *Streptococcus macedonicus*, which is a member of this specific complex, has been suggested to be adapted to milk and it has been hypothesized that it could be non pathogenic. These assumptions were based on the fact that the primary ecological niche of *S. macedonicus* appears to be naturally fermented foods, mostly of dairy origin similarly to *S. thermophilus*[13]. Initial *in vitro* and *in vivo* evaluation did not support virulence of *S. macedonicus* ACA-DC 198 [14]. PCR and Southern blotting analyses indicated the absence of several *Streptococcus pyogenes* pathogenicity genes. In addition, oral administration of the organism at high dosages (8.9 log cfu daily) for an extended period of time (12 weeks) to mice did not result in any observable adverse effects including inflammation in the stomach or translocation from the GIT to the organs of the animals [14]. Moreover, strains of *S. macedonicus* have been shown to present important technological properties of industrial cultures like the production of texturizing exopolysaccharides and anti-clostridial bacteriocins [13].

*Streptococcus macedonicus* was originally isolated from traditional Greek Kasseri cheese [15] and it is phylogenetically related to *Streptococcus gallolyticus* subsp. *gallolyticus* and *Streptococcus pasteurianus* (formerly known as *S. bovis* biotypes I and II.2, respectively), as well as to *Streptococcus infantarius* (formerly known as *S. bovis* biotype II.1). The inclusion of *S. macedonicus* and *S. pasteurianus* as subspecies of *S. gallolyticus* subsp. *gallolyticus* (from this point on *S. gallolyticus*) has been previously suggested [16], but this taxonomic reappraisal has not been formally accepted so far [17]. *Streptococcus gallolyticus* and *S. pasteurianus* are considered pathogenic. Preliminary investigations concerning the mechanisms by which *S. gallolyticus* causes endocarditis indicated that *S. macedonicus* may lack at least some of the pathogenic determinants implicated in this disease [18,19]. Furthermore, the recent study of the genome of *S. infantarius* subsp. *infantarius* CJ18 (from this point on *S. infantarius*) isolated from spontaneously fermented camel milk in Africa has indicated strain-dependent traits of adaptation to the dairy environment despite the fact that the species is considered as a putative pathogen [20]. Overall, the presence in fermented foods of SBSEC species with a currently unresolved pathogenicity status, such as *S. macedonicus* and *S. infantarius*, may represent an underestimated cause of concern in terms of food safety and public health, which needs to be addressed.

Here we present the first complete genome sequence of *S. macedonicus* in order to shed light on the biology of the species. We are particularly interested in assessing niche adaptation and in investigating the pathogenic potential of the strain analyzed based on comparative genomics against other complete genomes within the SBSEC. This is an important step to rationally deduce whether the bacterium is safe to be used as a starter or if extra technological measures are needed to avoid its presence in food fermentations.

## Results and discussion

### General features of *Streptococcus macedonicus* ACA-DC 198 genome

**Figure 1:**
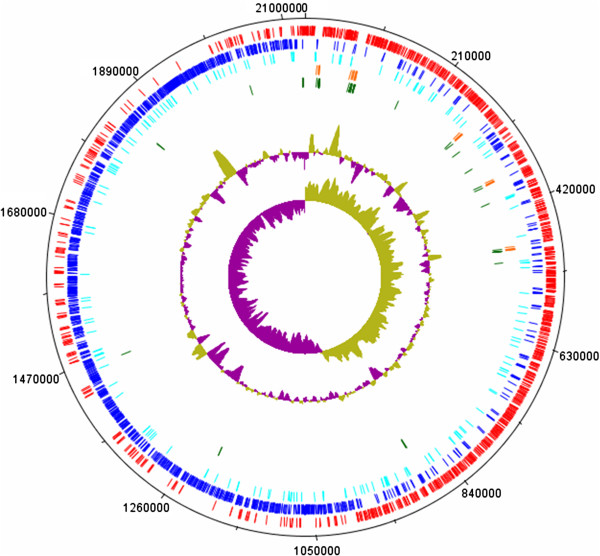
**The circular map of the genome of *Streptococcus macedonicus *ACA-DC 198.** Genomic features appearing from the periphery to the centre of the map: 1. Forward CDSs (red); 2. Reverse CDSs (blue); 3. Putative pseudogenes (cyan); 4. rRNA genes (orange); 5. tRNA genes (green); 6.% GC plot; 7. GC skew.

The circular chromosome of *S. macedonicus* ACA-DC 198 consists of 2,130,034 bp (Figure  1) with a G + C content of 37.6%, which is among the lowest values within the available complete streptococcal genomes (39.3% ± 1.7%, n = 95 by May 2013). A total of 2,192 protein coding DNA sequences (CDSs) were annotated, covering 87.3% of the *S. macedonicus* chromosome. Of these, 192 were identified as putative pseudogenes according to GenePRIMP [21] analysis followed by manual curation. The bacterium also carries 18 rRNA genes organized in 5 clusters co-localized with most of the 70 tRNA genes. The *S. macedonicus* genome was found to be 220–232 kb smaller and only 30 kb larger than the genomes of *S. gallolyticus* and *S. pasteurianus*, respectively. *Streptococcus infantarius* has one of the smallest genome sizes within the SBSEC reported up to now (i.e. 141 kb smaller than that of *S. macedonicus*). The percentage of potential pseudogenes in *S. macedonicus* was 8.7%, in *S. pasteurianus* 7.7% and in *S. infantarius* 4.9%. In contrast, the percentage of pseudogenes in at least two *S. gallolyticus* strains (i.e. strains UCN34 and ATCC 43143) has been found to be 2.1% or less. This analysis is in accordance with previous findings [9,22]. Based on the close phylogenetic relationship among the four species, these observations suggest that the genome of *S. macedonicus*, as well as those of *S. pasteurianus* and *S. infantarius* may be evolving under selective pressures that allow gene loss events and genome decay processes when compared to the *S. gallolyticus* genomes.

### Whole genome phylogeny, comparative genomics, and core genome analysis

A phylogenetic tree based on the currently available complete streptococcal genome sequences was constructed using the EDGAR software [23]. On this tree, *S. gallolyticus*, *S. macedonicus*, *S. pasteurianus,* as well as *S. infantarius* formed a single, monophyletic branch, providing strong evidence for the taxonomic integrity of the SBSEC (Additional file 1: Figure S1).

**Figure 2:**
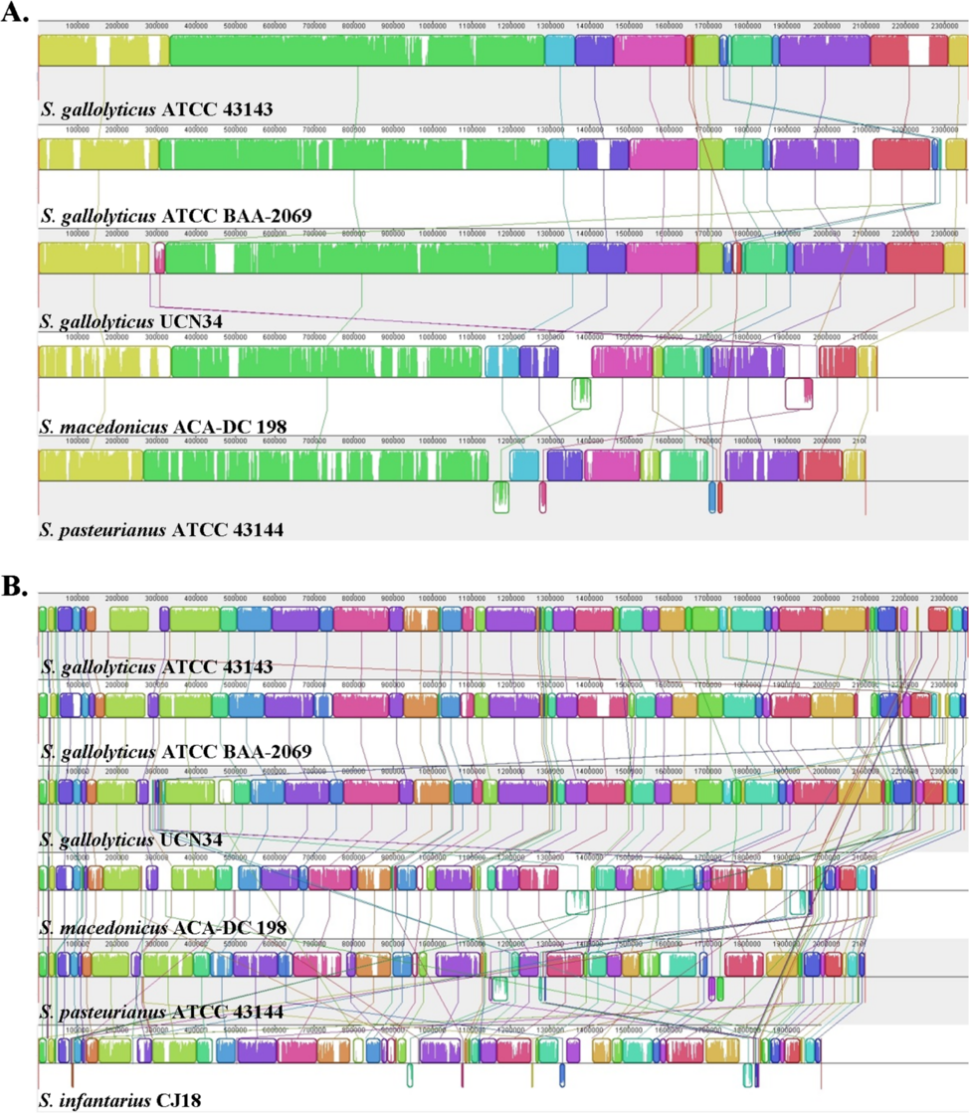
**Chromosome alignments of the *Streptococcus bovis*/*Streptococcus equinus *complex members as calculated by progressiveMauve.** Chromosome alignments among *Streptococcus gallolyticus*, *Streptococcus macedonicus* and *Streptococcus pasteurianus***(A)** and all the aforementioned streptococci and *Streptococcus infantarius***(B)**. Local collinear blocks (LCBs) of conserved sequences among the strains are represented by rectangles of the same colour. Connecting lines can be used to visualize synteny or rearrangement. LCBs positioned above or under the chromosome (black line) correspond to the forward and reverse orientation, respectively. The level of conservation is equivalent to the level of vertical colour filling within the LCBs (e.g. white regions are strain-specific). Sequences not placed within an LCB are unique for the particular strain.

**Figure 3:**
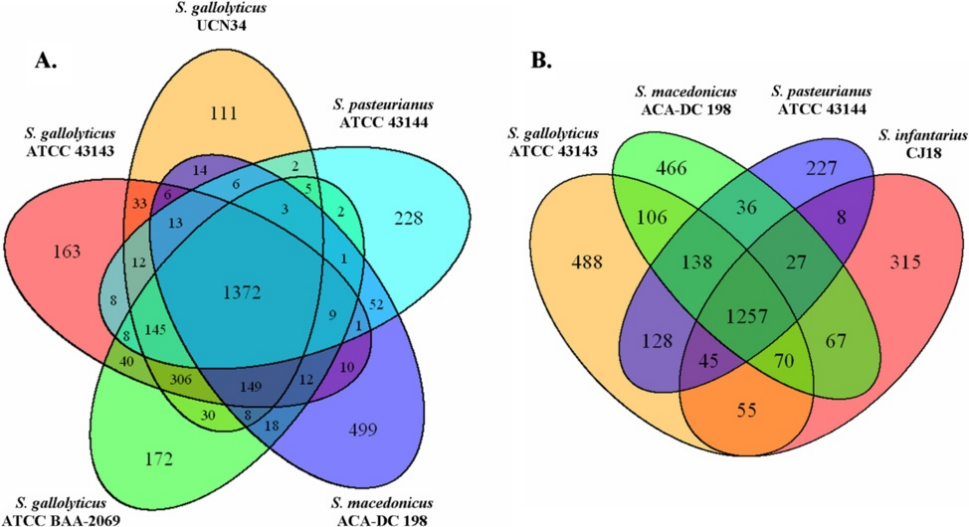
**Core genome analysis of members of the *Streptococcus bovis*/*Streptococcus equinus *complex.** Whole CDS Venn diagrams of *Streptococcus gallolyticus*, *Streptococcus macedonicus* and *Streptococcus pasteurianus***(A)** or *Streptococcus gallolyticus*, *Streptococcus infantarius*, *Streptococcus macedonicus* and *Streptococcus pasteurianus***(B)**. In **(B)***Streptococcus gallolyticus* ATCC 43143 was selected as a representative of the *S. gallolyticus* species, since it has the longest genome size among the three sequenced strains.

Subsequently, full chromosome alignments were performed using progressiveMAUVE [24]. The analysis revealed a mosaic pattern of homology organized in local collinear blocks (LCBs) among *S. gallolyticus*, *S. macedonicus* and *S. pasteurianus* (Figure  2A). Evidently, a significant portion of the genetic information has been overall conserved, as the majority of the LCBs are shared by all species. In addition, chromosomal rearrangements seem to have been rather minimal, as the number of LCBs showing a change in relative genomic position among the strains was low and their length short. Nevertheless, numerous differences were also detected. Some LCBs were common only among some of the strains, while some regions were identified as strain-specific (and hence not included within an LCB). The presence of such strain-specific regions suggests that, in addition to gene loss mentioned earlier, gene acquisition events mediated by horizontal gene transfer (HGT) may have played a role during the evolution of the three species (see below). Interestingly, the inclusion of the *S. infantarius* genome in the MAUVE analysis resulted in an increased number of LCBs with a decreased average length. As the level of sequence conservation of individual LCBs among the four species remains relatively high, this observation suggests that specific genome structure reorganization events occurred specifically in *S. infantarius* (Figure  2B). Analysis with the EDGAR software revealed a core genome of only 1,372 orthologous genes based on the sequence and the current annotation of *S. gallolyticus*, *S. pasteurianus* and *S. macedonicus* (Figure  3A, Additional file 2: Table S1) [23]. Once more, inclusion of *S. infantarius* increased the diversity, resulting in reduction of the core genome by more than 100 genes among the four species (Figure  3B, Additional file 3: Table S2). The significant percentage of variable genes within the four SBSEC species may underpin their adaptation to specific environments.

### Genes involved in the survival in the GIT

It has been established that *S. gallolyticus* displays the notable ability to accumulate and metabolize a broad range of complex carbohydrates from plants when compared to other streptococci [25]. The necessity for this repertoire of carbohydrate-degrading activities has been considered to reflect the adaptation of *S. gallolyticus* to the rumen of herbivores [22,25]. Preliminary analysis indicated that at least some of the relevant genes are either entirely absent or they have been converted into pseudogenes in the genomes of *S. macedonicus*, *S. pasteurianus* and *S. infantarius* (Table  1). The presence of pseudogenes related to carbohydrate metabolism reinforces the notion that *S. macedonicus*, *S. pasteurianus* and *S. infantarius* have undergone genome decay processes during adaptation to their ecological niches. The entire glycobiome of the SBSEC members was further analyzed based on the data available in the CAZy database (Additional file 4: Table S3) [26]. Important differences in the distribution of enzymes among the SBSEC members were observed for all CAZy categories including glycoside hydrolases (GHs), glycosyl tranferases, polysaccharide lyases, carbohydrate esterases and carbohydrate-binding modules (CBMs). *Streptococcus macedonicus* and *Streptococcus infantarius* had the smallest glycobiome within the SBSEC. The two strains had only 24 and 23 GHs, while the rest SBSEC members had more than 40. Given that most of these GHs are potentially involved in plant and dietary carbohydrate catabolism (e.g. GH1, GH3, GH13, GH36 etc.) [27], it could be hypothesized that *S. macedonicus* and *S. infantarius* have a diminished necessity for such enzymes in their ecological niche. *Streptococcus pasteurianus* had the highest number of GHs, some of which were unique among SBSEC (i.e. GH35, GH78, GH79, GH85, GH92, GH125). This observation indicates differences in the range of carbohydrates the strain is able to catabolize in comparison to the other members of the complex. Interestingly, none of the SBSEC members were found to carry GHs that are implicated in the degradation of host derived oligosaccharides (e.g. GH33 and GH98) [27]. In contrast, *Streptococcus gallolyticus* strains, *S. macedonicus* and *S. infantarius* had hits in the CBM41 family, while *S. pasteurianus* in the CBM32 family, both of which have been associated with the recognition of host glycans [27,28].

**Table 1:** **Genes in the *Streptococcus bovis*/*Streptococcus equinus *** **complex potentially involved in adaptation to the rumen**

**Function**	**Gene**	***S. gallolyticus *** **UCN34**	***S. gallolyticus *** **ATCC 43143**	***S. gallolyticus *** **ATCC BAA-2069**	***S. pasteurianus *** **ATCC 43144**	***S. macedonicus *** **ACA-DC 198**	***S. infantarius *** **CJ18**
Pullulanase	- (a)	GALLO_1462	SGGB_1458	SGGBAA2069_c14850	SGPB_1362 (t)	SMA_1464 (s)	Sinf_1270
						SMA_1465 (s)	
Pullulanase	-	GALLO_0781	SGGB_0764	SGGBAA2069_c07530	-	SMA_0719 (p)	-
						*SMA_0720* (r)	
						SMA_0721 (p)	
α-amylase, neopullulanase	-	GALLO_0753	SGGB_0736	SGGBAA2069_c07260	-	-	-
Fructan hydrolase	*fruA*	GALLO_0112	SGGB_0110	SGGBAA2069_c01280	-	-	-
Beta-1,4-endoglucanase V (cellulase)	-	GALLO_0330	SGGB_0358	SGGBAA2069_c03180	-	-	-
Cinnamoyl ester hydrolase	*cinA*	GALLO_0140	SGGB_0137	SGGBAA2069_c01580	-	-	-
Mannanase	-	GALLO_0162	SGGB_0206	SGGBAA2069_c01800	-	-	Sinf_0174 (p)
Endo-beta-1,4-galactanase	-	GALLO_0189	SGGB_0233	SGGBAA2069_c02070	SGPB_0176	SMA_0214 (p)	Sinf_0197 (p)
Pectate lyase	-	GALLO_1577	SGGB_1576	SGGBAA2069_c16050	-	-	Sinf_1418
Pectate lyase	-	GALLO_1578	SGGB_1577	SGGBAA2069_c16060	SGPB_1461 (p)	SMA_1582 (p)	-
						SMA_1583 (s)	
						SMA_1584 (s)	
Malate transporter	*mleP*	GALLO_2048	SGGB_2031	SGGBAA2069_c20060	SGPB_1855	SMA_1945	Sinf_1750
Malate dehydrogenase	*mleS*	GALLO_2049	SGGB_2032	SGGBAA2069_c20070	SGPB_1856	SMA_1946	Sinf_1751
PTS system, mannitol-specific IIBC component	*mtlA*	GALLO_0993	SGGB_0982	SGGBAA2069_c09680	-	SMA_0905 (p)	-
Mannitol operon transcriptional antiterminator	*mtlR*	GALLO_0994	SGGB_0983	SGGBAA2069_c09690	-	SMA_0906 (p)	-
						*SMA_0907*	
						*SMA_0908*	
						SMA_0909	
						SMA_0910	
						SMA_0911	
						SMA_0912	
						SMA_0913	
						SMA_0914	
						SMA_0915	
						*SMA_0916*	
						SMA_0917	
PTS system, mannitol-specific IIA component	*mtlF*	GALLO_0995	SGGB_0984	SGGBAA2069_c09700	-	-	-
Mannitol-1-phosphate 5-dehydrogenase	*mtlD*	GALLO_0996	SGGB_0985	SGGBAA2069_c09710	-	-	-
α-amylase	-	GALLO_0757	SGGB_0740	SGGBAA2069_c07300	-	-	-
α-amylase	*amyE*	GALLO_1632	SGGB_1646	SGGBAA2069_c16600	SGPB_1505 (p)	SMA_1612 (t)	Sinf_1443
α-amylase	-	GALLO_1043	SGGB_1033	SGGBAA2069_c10200	SGPB_0905	SMA_0972	Sinf_0846
tannase	*tanA*	GALLO_0933	SGGB_0917	SGGBAA2069_c09070 (s)	-	-	-
				SGGBAA2069_c09080 (s)			
Tannase (similar to tanA)	-	GALLO_1609	SGGB_1624	SGGBAA2069_c16370	-	-	-
Phenolic acid decarboxylase	*padC*	GALLO_2106	SGGB_2089	SGGBAA2069_c21040	SGPB_1899	SMA_2074	-
Carboxymuconolactone decarboxylase	-	GALLO_0906	SGGB_0891	SGGBAA2069_c08850	SGPB_0775	-	-
Bile salt hydrolase	*bsh*	GALLO_0818	SGGB_0803	SGGBAA2069_c07920	SGPB_0678	SMA_0753 (p)	Sinf_0639

(a) Not found; (t) Truncated; (s) Split CDSs corresponding to fragments of the original gene not yet characterized as pseudogenes; (p) Pseudogenes; (r) Transposase genes in italics.

Furthermore, *S. gallolyticus* can detoxify toxic compounds met in the rumen and other environments. Again, *S. macedonicus, S. pasteurianus* and *S. infantarius* miss some of the genes involved in detoxification (Table  1). None of them carry genes for tannin hydrolysis similar to GALLO_0933 or GALLO_1609. The potential to degrade additional phenolic compounds like gallic acid seems to be comparable between *S. gallolyticus* and *S. pasteurianus*. In contrast, *S. infantarius* has no orthologs of either PadC (GALLO_2106) or GALLO_0906, i.e. the two gallic acid decarboxylases found in *S. gallolyticus* UCN34, while *S. macedonicus* has retained only PadC. Furthermore, the *bsh* gene (GALLO_0818), coding for a bile salt hydrolase, is present in all four species with the exception of *S. macedonicus*, in which it appears as a pseudogene. Thus, our findings clearly suggest that not only *S. macedonicus*, but also *S. pasteurianus* and *S. infantarius* have deviated from *S. gallolyticus* in their potential to cope with the harsh environment of the GIT of herbivores.

### Genes involved in the growth in milk or dairy products

Dairy LAB are considered fastidious microorganisms due to their adaptation to growth in milk that is particularly nutritious by nature. Lactose and milk proteins (both caseins and whey proteins) are characteristic of the dairy environment. LAB are able to ferment lactose to lactic acid and they have evolved a proteolytic system for the degradation of milk proteins down to amino acids [1,29].

All SBSEC species are able to utilize lactose and to catabolize galactose. Sequence similarity searches revealed a gene cluster (SMA_0197 – SMA_0211) dedicated to lactose metabolism with a unique organization in SBSEC when compared to those previously reported for other LAB (Table  2). The typical sequence of *lac* genes is interrupted in the majority of SBSEC strains by genes coding for the IIA, IIB and IIC components of a PEP-PTS (SMA_0202 – SMA _0204). Annotation of this PEP-PTS varies among the SBSEC species/strains and for this reason functional analysis is required to properly determine its exact function. In contrast to other SBSEC species, these three PTS genes are absent from *S. infantarius*. The lactose-specific PTS found at the end of the *lac* gene cluster (SMA_0206 – SMA _0210) is also inactivated in *S. infantarius* through disruption of the *lacT* antiterminator gene by transposases [20]. Interestingly, the *lac* gene cluster in *S. macedonicus* contains two 6-phospho-beta-galactosidase (*lacG*) genes that may be indicative of adaptation of this particular species to milk. Galactose can also be catabolized through the Leloir pathway and a *galRKTE* operon coding for the relevant enzymes was previously determined in *S. infantarius*[30]. The *gal* operon is conserved in all SBSEC species analyzed here (Table  2).

**Table 2:** **Genes in the *Streptococcus bovis*/*Streptococcus equinus *** **complex potentially involved in lactose and galactose metabolism**

**Function**	**Gene**	***S. gallolyticus *** **UCN34**	***S. gallolyticus *** **ATCC 43143**	***S. gallolyticus *** **ATCC BAA-2069**	***S. pasteurianus *** **ATCC 43144**	***S. macedonicus *** **ACA-DC 198**	***S. infantarius *** **CJ18**
Lactose-specific PTS system repressor	*lacR*	GALLO_0176	SGGB_0220	SGGBAA2069_c01940	SGPB_0163	SMA_0197	Sinf_0181
Galactose-6-phosphate isomerase, LacA subunit	*lacA*	GALLO_0177	SGGB_0221	SGGBAA2069_c01950	SGPB_0164	SMA_0198	Sinf_0182
Galactose-6-phosphate isomerase, LacB subunit	*lacB1*	GALLO_0178	SGGB_0222	SGGBAA2069_c01960	SGPB_0165	SMA_0199	Sinf_0183
Tagatose-6-phosphate kinase	*lacC*	GALLO_0179	SGGB_0223	SGGBAA2069_c01970	SGPB_0166	SMA_0200	Sinf_0184
Tagatose 1,6-diphosphate aldolase	*lacD2*	GALLO_0180	SGGB_0224	SGGBAA2069_c01980	SGPB_0167	SMA_0201	Sinf_0185
Putative PTS system, IIA component	- (a)	GALLO_0181	SGGB_0225	SGGBAA2069_c01990	SGPB_0168	SMA_0202	-
Putative PTS system, IIB component	-	GALLO_0182	SGGB_0226	SGGBAA2069_c02000	SGPB_0169	SMA_0203	-
Putative PTS system, IIC component	-	GALLO_0183	SGGB_0227	SGGBAA2069_c02010	SGPB_0170	SMA_0204	-
Aldose 1-epimerase	*lacX*	GALLO_0184	SGGB_0228	SGGBAA2069_c02020	SGPB_0171	SMA_0205	Sinf_0186
Transcriptional antiterminator	*lacT*	GALLO_0185	SGGB_0229	SGGBAA2069_c02030	SGPB_0172	SMA_0206	Sinf_0187 (p)
							*Sinf_0188* (r)
							*Sinf_0189*
							Sinf_0190 (p)
6-phospho-beta-galactosidase	*lacG*	GALLO_0186	SGGB_0230	SGGBAA2069_c02040	SGPB_0173	SMA_0207	-
Transcriptional antiterminator	*lacT*	-	-	-	-	SMA_0208 (p)	-
Lactose-specific PTS system, IIA component	*lacF*	GALLO_0187	SGGB_0231	SGGBAA2069_c02050	SGPB_0174	SMA_0209	Sinf_0191
Lactose-specific PTS system, IIBC component	*lacE*	GALLO_0188	SGGB_0232	SGGBAA2069_c02060	SGPB_0175	SMA_0210	Sinf_0192
6-phospho-beta-galactosidase	*lacG2*	-	-	-	-	SMA_0211	Sinf_0193 (p)
							*Sinf_0194*
							Sinf_0195 (p)
Galactose repressor	*galR*	GALLO_0197	SGGB_0241	SGGBAA2069_c02150	SGPB_0184	SMA_0222	Sinf_0205
Galactokinase	*galK*	GALLO_0198	SGGB_0242	SGGBAA2069_c02160	SGPB_0185	SMA_0223	Sinf_0206
Galactose-1-P-uridyl transferase	*galT*	GALLO_0199	SGGB_0243	SGGBAA2069_c02170	SGPB_0186	SMA_0224	Sinf_0207
UDP-glucose 4-epimerase	*galE*	GALLO_0200	SGGB_0244	SGGBAA2069_c02180	SGPB_0187	SMA_0225	Sinf_0208
Beta-galactosidase	*lacZ*	-	-	-	SGPB_0344	-	-
Glucokinase	*glcK*	GALLO_0594	SGGB_0562	SGGBAA2069_c05300	SGPB_0467	SMA_0546	Sinf_0470
Beta-galactosidase	*lacZ*	-	-	-	-	-	Sinf_0935
Lactose and galactose permease	*lacS*	-	-	-	-	-	Sinf_0936
Aldose 1-epimerase	*galM*	-	-	-	-	-	Sinf_0937
UDP-glucose 4-epimerase	*galE1*	-	-	-	-	-	Sinf_0938
Galactose-1-P-uridyl transferase	*galT*	-	-	-	-	-	Sinf_0939 (p)
UDP-glucose 4-epimerase	*lacS*	-	-	-	-	-	Sinf_1514
Aldose 1-epimerase	*lacX*	-	-	-	-	SMA_1156	-
6-phospho-beta-galactosidase	*lacG2*	-	-	-	-	SMA_1157	-
Lactose-specific PTS system, IIBC component	*lacE*	-	-	-	-	SMA_1158	-
Lactose-specific PTS system, IIA component	*lacF*	-	-	-	-	SMA_1159	-
Tagatose 1,6-diphosphate aldolase	*lacD*	-	-	-	-	SMA_1160	-
Tagatose-6-phosphate kinase	*lacC*	-	-	-	-	SMA_1161	-
Galactose-6-phosphate isomerase, LacB subunit	*lacB*	-	-	-	-	SMA_1162	-
Galactose-6-phosphate isomerase, LacA subunit	*lacA1*	-	-	-	-	SMA_1163	-
Glucokinase	*glcK*	-	-	-	-	SMA_1164	-
Lactose phosphotransferase system repressor	*lacR*	-	-	-	-	SMA_1165	-
Transcription antiterminator	*lacT*	GALLO_1046	SGGB_1036	SGGBAA2069_c10230	SGPB_0907	-	-
Lactose-specific PTS system, IIA component	*lacF*	GALLO_1047	SGGB_1037	SGGBAA2069_c10240	SGPB_0908	-	-
Lactose-specific PTS system, IIBC component	*lacE*	GALLO_1048	SGGB_1038	SGGBAA2069_c10250	SGPB_0909	-	-
Phospho-beta-galactosidase	*lacG*	GALLO_1049	SGGB_1039	SGGBAA2069_c10260	SGPB_0910	-	-
Aldose 1-epimerase	*galM*	GALLO_0137	SGGB_0134	SGGBAA2069_c01550	SGPB_0130	-	-
UDP-glucose 4-epimerase	*galE1*	GALLO_0728	SGGB_0709	SGGBAA2069_c06910	SGPB_0601	-	-

(a) Not found; (p) Pseudogenes; (r) Transposase genes in italics.

A partial *gal*-*lac* operon *galT*(truncated)/*galE1M*/*lacSZ* with high sequence identity to *S. thermophilus* is also present in the genome of *S. infantarius*[30]. It has been demonstrated that the lactose and galactose permease (*lacS*) and the β-galactosidase (*lacZ*) are responsible for the uptake and initial hydrolysis of lactose in *S. infantarius* in a manner similar to that employed by *S. thermophilus*[20]. This *gal*-*lac* operon of *S. infantarius* is missing from the other SBSEC strains as a whole. A LacZ ortholog (SGPB_0344) is only present in *S. pasteurianus* and dispersed *galE* and *galM* genes can be found in the *S. gallolyticus* and *S. pasteurianus* genomes. Similarly to the presence of the extra *gal*-*lac* operon in *S. infantarius*, we detected a second *lac* gene cluster in *S. macedonicus* (SMA_1156 – SMA_1165), also suggesting adaptation to the milk environment. This second gene cluster is solely present in *S. macedonicus* and not in any other SBSEC member. Surprisingly, an additional *lacTFEG* region coding for a complete lactose PEP-PTS and a 6-phospho-beta-galactosidase is present in the genomes of *S. gallolyticus* and *S. pasteurianus*. This is an unexpected finding since *S. gallolyticus* and S*. pasteurianus* have hardly ever been related to milk up to now [9].

We then investigated the proteolytic system of *S. macedonicus* and the rest of the SBSEC members adapting the scheme previously described by Liu and co-workers (i.e. excluding housekeeping proteases or proteases involved in specific cellular processes other than the acquisition of amino acids) [29]. In milk, casein utilization by LAB is initiated after hydrolysis by a cell-envelope associated proteinase (CEP) releasing oligopeptides. The oligopeptides are then transferred intracellularly via specialized peptide transport systems where they are systematically degraded into amino acids by an array of intracellular peptidases. The four species have essentially the same proteolytic system, albeit showing some differences (Table  3). None of them has a typical PrtP CEP, but *S. gallolyticus* and *S. infantarius* carry a lactocepin coding gene. The lactocepin of the SBSEC shows ≥ 63% sequence similarity to the PrtS CEP involved in the degradation of milk proteins in *S. thermophilus*[31,32]. The exact role of lactocepin in SBSEC species needs to be experimentally examined. SBSEC strains like *S. macedonicus* may require CEP activity to be provided by other bacteria when growing in milk. This is a common strategy of nonstarter LAB that rely on starter CEP-producing strains for casein hydrolysis [33]. *Streptococcus infantarius* carries two oligopeptide transport systems (Opp) [20], but all the other SBSEC species have only one such system. All SBSEC strains own a proton motive force (PMF)-driven DtpT transporter for the transport of di- and tri-peptides and they all possess an entire repertoire of proteolytic enzymes including endopeptidases, general aminopeptidases and specialized peptidases (Table  3). They only lack enzymes of the PepE/PepG (endopeptidases) and the PepI/PepR/PepL (proline peptidases) superfamilies in accordance to previous observations for streptococci and lactococci [29]. The conservation of this proteolytic system among streptococci in the SBSEC despite their presumed adaptation to different ecological niches [20,22,25] indicates that it may somehow be essential. Furthermore, *S. macedonicus* and the other SBSEC members are autotrophs for several amino acids (data not shown) and only *S. pasteurianus* has been reported to be unable to synthesize tryptophan [22]. Thus, the preservation of an entire proteolytic system by SBSEC members while retaining the ability to synthesize most, if not all, amino acids is puzzling, especially when considering that some of them have obviously undergone extensive genome decay processes. It could be hypothesized that this property of SBSEC species may provide a competitive advantage in poor environments, but this needs to be further investigated.

**Table 3:** **Genes in the *Streptococcus bovis*/*Streptococcus equinus *** **complex potentially involved in proteolysis of milk proteins**

**Function**	**Gene**	***S. gallolyticus *** **UCN34**	***S. gallolyticus *** **ATCC 43143**	***S. gallolyticus *** **ATCC BAA-2069**	***S. pasteurianus *** **ATCC 43144**	***S. macedonicus *** **ACA-DC 198**	***S*. *infantarius *** **CJ18**
Lactocepin	*prtS*	GALLO_0748	SGGB_0730	SGGBAA2069_c07210	-	-	Sinf_0588
Oligopeptide ABC transporter, substrate-binding protein	*oppA*	GALLO_0324	SGGB_0352	SGGBAA2069_c03120	SGPB_0276	SMA_0353	Sinf_0305
		GALLO_1412	SGGB_1406	SGGBAA2069_c14340	SGPB_1328	SMA_1347	Sinf_1225
		GALLO_1413	SGGB_1407	SGGBAA2069_c14350			Sinf_1226
							Sinf_1825
Oligopeptide ABC transporter, permease protein	*oppB*	GALLO_0325	SGGB_0353	SGGBAA2069_c03130	SGPB_0277	SMA_0354	Sinf_0306
							Sinf_1824
Oligopeptide ABC transporter, permease protein	*oppC*	GALLO_0326	SGGB_0354	SGGBAA2069_c03140	SGPB_0278	SMA_0355	Sinf_0307
							Sinf_1823
Oligopeptide ABC transporter, ATP-binding protein	*oppD*	GALLO_0327	SGGB_0355	SGGBAA2069_c03150	SGPB_0279	SMA_0356	Sinf_0308
							Sinf_1822
Oligopeptide ABC transporter, ATP-binding protein	*oppF*	GALLO_0328	SGGB_0356	SGGBAA2069_c03160	SGPB_0280	SMA_0357	Sinf_0309
							Sinf_1821
Dipeptide/tripeptide permease	*dtpT*	GALLO_0638	SGGB_0613	SGGBAA2069_c05810	SGPB_0507	SMA_0600	Sinf_0519
Cysteine aminopeptidase C	*pepC*	GALLO_0478	SGGB_0452	SGGBAA2069_c04140	SGPB_0379	SMA_0442	Sinf_0388
Aminopeptidase N	*pepN*	GALLO_1143	SGGB_1134	SGGBAA2069_c11310	SGPB_1002	SMA_1066	Sinf_0984
Methionine aminopeptidase	*pepM*	GALLO_0775	SGGB_0758	SGGBAA2069_c07470	SGPB_0642	SMA_0713	Sinf_0604
Glutamyl aminopeptidase	*pepA*	GALLO_0101	SGGB_0101	SGGBAA2069_c01190	SGPB_0100	SMA_0113	Sinf_0111
		GALLO_0151	SGGB_0195	SGGBAA2069_c01680	SGPB_0141		
Endopeptidase	*pepO*	GALLO_2172	SGGB_2204	SGGBAA2069_c21680	SGPB_1933	SMA_2096	Sinf_1874
Oligoendopeptidase	*pepF*	GALLO_0669	SGGB_0651	SGGBAA2069_c06210	SGPB_0551	SMA_0630	Sinf_0554
		GALLO_1516	SGGB_1511	SGGBAA2069_c15390	SGPB_1410	SMA_1526	Sinf_1335
Dipeptidase	*pepD*	GALLO_0732	SGGB_0713	SGGBAA2069_c06950	SGPB_0605	SMA_0668	Sinf_1301
Xaa-His dipeptidase	*pepV*	GALLO_0931	SGGB_0915	SGGBAA2069_c09050	SGPB_0797	SMA_0836	Sinf_0699
Peptidase T	*pepT*	GALLO_1366	SGGB_1360	SGGBAA2069_c13560	SGPB_1287	SMA_1297	Sinf_1183
X-prolyl-dipeptidyl aminopeptidase	*pepX*	GALLO_1959	SGGB_1942	SGGBAA2069_c19090	SGPB_1791	SMA_1862	Sinf_1676
Aminopeptidase P	*pepP*	GALLO_1901	SGGB_1885	SGGBAA2069_c18550	SGPB_1732	SMA_1811	Sinf_1626
Xaa-proline dipeptidase	*pepQ*	GALLO_1583	SGGB_1582	SGGBAA2069_c16110	SGPB_1466	SMA_1589	Sinf_1424

Apart from amino acids, *S. gallolyticus* UCN34 also carries complete pathways for the synthesis of a number of vitamins including riboflavin, nicotine amide, pantothenate, pyridoxine, and folic acid, while the biosynthetic pathways for biotin and thiamine are partial [25]. The genes potentially involved in the *de novo* biosynthesis of pyridoxine in the SBSEC strains were determined based on the respective pathway of *S. pneumoniae* D39 [34]. The corresponding loci are conserved among *S. gallolyticus* strains but once more *S. macedonicus*, *S. pasteurianus* and *S. infantarius* appear to have undergone a heterogeneous gene loss process, indicating the necessity for exogenous supply of some of these vitamins (Table  4). For example, *S. macedonicus* misses the *bioBDY, panBCD* and *ribDEAH* loci involved in the biosynthesis of biotin, pantothenate and riboflavin, respectively. In addition, the presence of pseudogenes or truncated/split genes may have disrupted the biosynthesis of pyridoxine, nicotine amide and thiamine through the routes analyzed here. It is not uncommon for LAB to be auxotrophic for several vitamins [35], though milk and other dairy products may contain all essential vitamins to sustain the growth of these microorgansims.

**Table 4:** **Genes in the *Streptococcus bovis*/*Streptococcus equinus *** **complex potentially involved in the biosynthesis of vitamins**

**Vitamin**	**Gene**	***S*. *gallolyticus *** **UCN34**	***S*. *gallolyticus *** **ATCC 43143**	***S. gallolyticus *** **ATCC BAA-2069**	***S. pasteurianus *** **ATCC 43144**	***S. macedonicus *** **ACA-DC 198**	***S*. *infantarius *** **CJ18**
Biotin (B8, partial)	*bioB*	GALLO_1916	SGGB_1900	SGGBAA2069_c18670	SGPB_1745	- (a)	-
	*bioD*	GALLO_1915	SGGB_1899	SGGBAA2069_c18660	SGPB_1744	-	-
	*bioY*	GALLO_1914	SGGB_1898	SGGBAA2069_c18650	SGPB_1743	-	-
	*pdxS*	GALLO_1189	SGGB_1183	SGGBAA2069_c11790	-	SMA_1105 (s)	Sinf_1022
						SMA_1106 (p)	
	*pdxT*	GALLO_1188	SGGB_1182	SGGBAA2069_c11780	-	SMA_1104	Sinf_1021
	*pdxR*	GALLO_1111	SGGB_1101	SGGBAA2069_c10980	SGPB_0968	SMA_1031	Sinf_0955
Folic acid (B9)	*folC*	GALLO_1233	SGGB_1227	SGGBAA2069_c12240	SGPB_1087	SMA_1137	Sinf_1067
	*folE*	GALLO_1232	SGGB_1226	SGGBAA2069_c12230	SGPB_1086	SMA_1136	Sinf_1066
	*folP*	GALLO_1231	SGGB_1225	SGGBAA2069_c12220	SGPB_1085	SMA_1135	Sinf_1065
	*folB*	GALLO_1230	SGGB_1224	SGGBAA2069_c12210	SGPB_1084	SMA_1134	Sinf_1064
	*folK*	GALLO_1229	SGGB_1223	SGGBAA2069_c12200	SGPB_1083	SMA_1133	Sinf_1063
	*folD*	GALLO_0622	SGGB_0594	SGGBAA2069_c05620	SGPB_0494	SMA_0581	Sinf_0503
Nicotine amide (NAD, B3)	*nadA*	GALLO_1937	SGGB_1920	SGGBAA2069_c18890	SGPB_1769	SMA_1844 (p)	Sinf_1655
	*nadB*	GALLO_1936	SGGB_1919	SGGBAA2069_c18880	SGPB_1768	SMA_1840 (s)	Sinf_1654
						SMA_1841 (s)	
						SMA_1842 (s)	
						SMA_1843 (p)	
	*nadC*	GALLO_1935	SGGB_1918	SGGBAA2069_c18870	SGPB_1767	SMA_1839	Sinf_1653
	*nadE*	GALLO_0477	SGGB_0451	SGGBAA2069_c04130	SGPB_0377 (p)	SMA_0441	Sinf_0387
					SGPB_0378 (p)		
Pantothenate (B5)	*panB*	GALLO_0161	SGGB_0205	SGGBAA2069_c01790	-	-	-
	*panC*	GALLO_0160	SGGB_0204	SGGBAA2069_c01780	-	-	Sinf_0173 (t)
	*panD*	GALLO_0159	SGGB_0203	SGGBAA2069_c01770	-	-	Sinf_0172
	*panE*	GALLO_0232	SGGB_0274	SGGBAA2069_c02470	SGPB_0217	SMA_0254	Sinf_0233 (p)
Riboflavin (B2)	*ribD*	GALLO_0692	SGGB_0673	SGGBAA2069_c06490	SGPB_0567	-	Sinf_0572
	*ribE*	GALLO_0693	SGGB_0674	SGGBAA2069_c06500	SGPB_0568	-	Sinf_0573
	*ribA*	GALLO_0694	SGGB_0675	SGGBAA2069_c06510	SGPB_0569	-	Sinf_0574
	*ribH*	GALLO_0695	SGGB_0676	SGGBAA2069_c06520	SGPB_0570	-	Sinf_0575
	*ribF*	GALLO_1160	SGGB_1152	SGGBAA2069_c11480	SGPB_1019	SMA_1086	Sinf_0999
Thiamine (B1, partial)	*tenA*	GALLO_1181	SGGB_1175	SGGBAA2069_c11710	SGPB_1039	-	Sinf_1014
	*thiE*	GALLO_1178	SGGB_1172	SGGBAA2069_c11680	SGPB_1036	SMA_1100 (t)	Sinf_1011
	*thiM*	GALLO_1179	SGGB_1173	SGGBAA2069_c11690	SGPB_1037	-	Sinf_1012
	*thiD*	GALLO_1180	SGGB_1174	SGGBAA2069_c11700	SGPB_1038	-	Sinf_1013
	*thiI*	GALLO_1346	SGGB_1341	SGGBAA2069_c13350	SGPB_1268	SMA_1273	Sinf_1163
	*thiN*	GALLO_2003	SGGB_1987	SGGBAA2069_c19580	SGPB_1830	SMA_1899	Sinf_1712

(a) Not found; (s) Split CDSs corresponding to fragments of the original gene not yet characterized as pseudogenes; (p) Pseudogenes; (t) Truncated.

### Genomic islands (GIs) and unique genes of *Streptococcus macedonicus*

GIs are sites of HGT that can uncover important features of the plasticity of a bacterial genome and they are primarily linked to gene gain processes. We used the IslandViewer application [36] to identify GIs of the SBSEC members in parallel. *Streptococcus macedonicus* had 14 predicted GIs with an average length of 18,109 bp corresponding to a total sequence of 253,523 bp or 11.9% the size of the bacterium’s genome (Additional file 5: Figure S2). This percentage of externally acquired DNA is higher compared to the other SBSEC members, in which it ranged from 8.8% in *S. gallolyticus* ATCC BAA-2069 down to 5.9% in *S. gallolyticus* UCN34.

As could be expected, the highest degree of sequence conservation among GIs was observed in the *S. gallolyticus* strains (e.g. *S. gallolyticus* UCN34 GIs 2, 6, 7, 8 and 9). When different SBSEC species were compared, a number of GIs were only partially conserved (e.g. *S. gallolyticus* UCN34 GIs 1, 3, 6, 7, 8 and 9). Unique GIs were also present in most genomes analyzed (e.g. *S. pasteurianus* GIs 2, 4, 6 and 8). Partially conserved GIs may be remnants of GIs acquired before speciation events in the SBSEC and their subsequent gene decay may be the result of adaptation to diverged ecological niches. The existence of unique GIs among the SBSEC species, whose acquisition must have been more recent (i.e. most probably after speciation), also points to the same direction. Furthermore, our analysis suggests that *S. macedonicus* shares stretches of GI sequences exclusively with *S. infantarius* among the SBSEC members (e.g. in *S. macedonicus* GIs 1, 4, 5, 6, 7, 8 and 14) in accordance with previous findings [20]. Potential donors of GI sequences were identified from best BLASTN hits showing sequence identity > 90%. In several instances sequence segments within *S. macedonicus* GIs may have derived from more than one donor (Additional file 6: Figure S3). Potential donors of the *S. macedonicus* GIs were *Streptococcus agalactiae*, *Streptococcus intermedius*, *Streptococcus suis*, *Streptococcus uberis, Enterococcus faecium*, *Lactococcus garvieae* and *Pediococcus pentosaceus.* Most importantly, *Lactococcus lactis* or *S. thermophilus* were found among these donors in 9 out of 14 *S. macedonicus* GIs and the same applies for *S. infantarius* in 6 out of 12 GIs. None of the GI sequences of the other SBSEC members could be linked to *L. lactis* or *S. thermophilus* apart from the *S. gallolyticus* ATCC BAA-2069 GI 6 that exhibited a 96% identity over an approximately 3 kb genomic region of *S. thermophilus* JIM 8232 (data not shown). These observations constitute additional evidence that *S. macedonicus* and *S. infantarius* are the only members of the complex that have extensively interacted with the dairy *L. lactis* and *S. thermophilus*.

We then calculated the unique genes (also referred here as singleton genes) of *S. macedonicus* against the other SBSEC species twice, taking or not into account the genome of *S. infantarius*. Results from singleton gene analysis using EDGAR [23] were manually curated to relieve the set from the high numbers of transposable elements. There was an important overlap between the list of genes found in GIs of *S. macedonicus* and the singleton genes (Additional file 7: Table S4 and Additional file 8: Table S5). Again, *S. macedonicus* and *S. infantarius* were found to share a number of genes that are absent from the other SBSEC genomes (Additional file 8: Table S5).

According to the aforementioned analysis *S. macedonicus* carries the complete biosynthetic pathways for two lantibiotic bacteriocins, i.e. the macedocin and the macedovicin peptides [37,38]. The presence of both antimicrobials can provide an additional link between *S. macedonicus* and the milk environment. Production of macedocin has been observed only in milk up to now and proteolytic fragments of casein may trigger biosynthesis of this peptide [39]. In addition, the entire macedovicin gene cluster is practically identical (99% sequence identity over the entire length of the ~9.8 kb cluster) to the respective clusters of thermophilin 1277 and bovicin HJ50 found in the dairy isolates *S. thermophilus* SBT1277 and *S. bovis* HJ50, respectively [37]. The locus seems to have spread among the three strains by HGT and their common dairy origin increases the possibility that this exchange of genetic material has taken place in milk [37].

Another evident characteristic of the *S. macedonicus* genome was the presence of multiple restriction modification (RM) systems among the singleton genes (Additional file 9: Figure S4). *Streptococcus macedonicus* possesses the highest number of RM systems within the SBSEC and it is the only member of the group with all three types of RM systems. A yet unresolved difference in the number and the type of RM systems between commensal and dairy LAB has been previously observed [40,41]. As mentioned earlier, phages are present in milk and dairy products often in high numbers [42] and traditional practices (e.g. backslopping) may promote the selection of phage resistant strains [40,41]. In *S. thermophilus* RM systems are considered as important technological traits [8] and it has been previously suggested that genes of the type III RM system may provide a signature for milk adaptation [40]. *Streptococcus macedonicus* has two type III RM systems, one of which is inactive since it consists of pseudogenes. The increased number of RM systems of *S. macedonicus* compared to the other SBSEC members suggests that it should be particularly competent in resisting invading DNA. These findings coincide with the fact that *S. macedonicus* carries the highest number of spacers in its CRISPR (clustered regularly interspaced short palindromic repeats) locus within the SBSEC (Additional file 10: Table S6). Furthermore, BLASTN analysis of the spacers in the *S. macedonicus* CRISPR revealed that four of them, namely spacers 3, 5, 17 and 18, had hits in *S. thermophilus* phages (e.g. phages O1205, 7201, Abc2, etc.), *S. thermophilus* plasmids (e.g. pER36) or *S. thermophilus* CRISPR spacer sequences (data not shown). In contrast, among the 140 spacers of the different CRISPR found in the other SBSEC species, only one had a hit in *L. lactis* phage 1706 (spacer 35 in the CRISPR of *S. pasteurianus*). According to these findings the occurrence of *S. macedonicus* in the same habitat as that of *S. thermophilus* can be supported.

In addition, *S. macedonicus* contains singleton genes – several copies in some instances – coding for proteins involved in the transport and homeostasis of metal ions (Additional file 7: Table S4 and Additional file 8: Table S5). Some of these genes are also shared by *S. infantarius*, but not all. These genes may play a role in the transport of copper (e.g. *copA* and *copB*), cadmium (e.g. *cadA* and *cadC*), manganese (e.g. *mntH*) and magnesium (e.g. SMA_2044). Copper and cadmium are of no evident biological role for *Lactobacillales*[43] and thus transport systems for such metals in *S. macedonicus* should be perceived as a protective mechanism towards their deleterious effects (e.g. through oxidative stress). The presence of metal transport genes has been previously reported in several LAB including *L. lactis* and *S. thermophilus* strains [43-48]. In our opinion the high number of metal transport associated genes in *S. macedonicus* was an unexpected observation and further investigation is required regarding their physiological relevance.

### Distribution of virulence factors (VFs) within species of the SBSEC

One of the main goals behind the genome sequencing of *S. macedonicus* was to clarify its pathogenic potential. Unfortunately, despite the well-known association of *S. bovis* with human disease, especially endocarditis and colon cancer, there is very little knowledge about the pathogenicity mechanisms employed by members of the SBSEC. In Table  5 we have gathered genes previously assigned as potential VFs in SBSEC. The available studies have shed some light on the ability of *S. gallolyticus* to colonize host tissues, a step that is considered as a prerequisite for the initiation of the infection by this bacterium. *Streptococcus gallolyticus* UCN34 contains three pilus gene clusters which may mediate binding to the extracellular matrix (ECM), similarly to the clinical isolate TX20005 whose genome is partially characterized [25,49]. The *pil1* and *pil3* of strain UCN34 have been found identical to the *acb-sbs7-srtC1* and *sbs15-sbs14-srtC3* loci of strain TX20005, respectively, but their additional predicted pilus gene cluster (i.e. *pil2* vs. *sbs12*-*sbs11*-*srtC2*) was only distantly related [25]. While all three strains of *S. gallolyticus* carry the three pilus loci (as found in strain UCN34), *S. macedonicus*, *S. pasteurianus* and *S. infantarius* carry only the *pil3* locus. Functional analysis indicated that *pil1* is a crucial factor of *S. gallolyticus* UCN34 for binding to ECM, especially to collagen [18]. The preference of *S. gallolyticus* to bind to collagen is of particular importance, since it may allow the adherence of the bacterium to the collagen-rich surfaces of damaged heart valves and (pre)cancerous sites [50]. Besides the pilus loci, additional MSCRAMM (microbial surface recognizing adhesive matrix molecules) proteins have been predicted in *S. gallolyticus,* most of which are either absent or preudogenes in *S. macedonicus*, *S. pasteurianus* and *S. infantarius* (Table  5) [49]. The cell surface protein antigen c (PAc) also appears exclusively in the *S. gallolyticus* genomes, sometimes in more than one copy. Only the surface-exposed histone-like protein A (HlpA) and the autolysin (AtlA) are universally conserved in the SBSEC. HlpA has been shown to be a major heparin-binding protein regulating the ability of *S. gallolyticus* adherence to the heparan sulfate proteoglycans at the colon tumor cell surface [51]. AtlA is a fibronectin-binding protein which is a VF of *S. mutans* associated with infective endocarditis [52]. Furthermore, *S. gallolyticus* UCN34 carries loci for the biosynthesis of insoluble glucan polymers from sucrose and the synthesis of hemicellulose [25]. Insoluble glucan polymers may contribute to feedlot bloat in cattle [25], while hemicellulose could play a role in biofilm formation [53]. It is possible that the production of these polymers may vary among strains of *S. gallolyticus* (Table  5). *Streptococcus macedonicus* is devoid of the biosynthetic gene cluster of glucan, while the hemicellulose synthesis operon seems to be comprised of pseudogenes. Similarly, *S. pasteurianus* and *S. infantarius* seem to be also unable to synthesize both sugar polymers, either due to full or partial absence of the genetic loci.

**Table 5:** **Genes in the *Streptococcus bovis*/*Streptococcus equinus *** **complex identified as putative virulence factors**

**Virulence factor**	**Gene**	***S. gallolyticus *** **UCN34**	***S*. *gallolyticus *** **ATCC 43143**	***S*. *gallolyticus *** **ATCC BAA-2069**	***S. pasteurianus *** **ATCC 43144**	***S. macedonicus *** **ACA-DC 198**	***S*. *infantarius *** **CJ18**
Pilus 1 (pil1)	*acb*	GALLO_2179	SGGB_2211	SGGBAA2069_c21760	- (a)	-	-
	*sbs7*	GALLO_2178	SGGB_2210	SGGBAA2069_c21750	SGPB_1938 (p)	-	-
	*srtC1*	GALLO_2177	SGGB_2209	SGGBAA2069_c21740	-	-	Sinf_1876
Pilus 2 (pil2)	*-*	GALLO_1570	SGGB_1568	SGGBAA2069_c15960	-	-	-
	*-*	GALLO_1569	SGGB_1567	SGGBAA2069_c15950	-	-	-
	*-*	GALLO_1568	SGGB_1566	SGGBAA2069_c15940	-	-	-
Pilus 3 (pil3)	*sbs15*	GALLO_2040	SGGB_2022	SGGBAA2069_c19980	SGPB_1847	SMA_1939	Sinf_1744
	*sbs14*	GALLO_2039	SGGB_2021	SGGBAA2069_c19970	SGPB_1846	SMA_1938	Sinf_1743
	*srtC3*	GALLO_2038	SGGB_2020	SGGBAA2069_c19960	SGPB_1845	SMA_1937	Sinf_1742
Cell envelope proteinase (lactocepin)	*sbs6*	GALLO_0748	SGGB_0730	SGGBAA2069_c07210	-	-	Sinf_0588
Fructan hydrolase	*sbs10*	GALLO_0112	SGGB_0110	SGGBAA2069_c01280	-	-	-
Collagen adhesin	*sbs13*	GALLO_2032	SGGB_2016	SGGBAA2069_c19910	SGPB_1839 (p)	SMA_1932 (s)	Sinf_1737 (p)
					SGPB_1840 (p)	SMA_1933 (p)	
						SMA_1934 (s)	
Collagen adhesin	*sbs16*	GALLO_0577	SGGB_0544	SGGBAA2069_c05110	-	-	-
Cell surface protein antigen C (PAc)	*-*	GALLO_1675	SGGB_0154	SGGBAA2069_c13880	-	-	-
			SGGB_1687	SGGBAA2069_c20560			
Surrface-exposed histone-like protein A	*hlpA*	GALLO_0636	SGGB_0611	SGGBAA2069_c05790	SGPB_0505	SMA_0597	Sinf_0517
Autolysin	*atlA*	GALLO_1368	SGGB_1362	SGGBAA2069_c13580	SGPB_1289	SMA_1299	Sinf_1186 (t)
Glucan biosynthesis gene cluster	*-*	GALLO_1052	-	SGGBAA2069_c10370	-	-	-
	*-*	GALLO_1053	SGGB_1042	SGGBAA2069_c10380	-	-	-
	*rggA*	GALLO_1054	SGGB_1043	SGGBAA2069_c10390	-	-	Sinf_0876
	*gtfA*	GALLO_1055	SGGB_1044	SGGBAA2069_c10400	-	-	Sinf_0877
	*rggB*	GALLO_1056	SGGB_1045	SGGBAA2069_c10410	-	-	-
	*gtfB*	GALLO_1057	SGGB_1046	SGGBAA2069_c10420	-	-	-
	*sbs2*/*gbpC*	GALLO_1058	SGGB_1047	SGGBAA2069_c10430	-	SMA_0989 (p)	-
						SMA_0990 (s)	
						SMA_0991 (s)	
Hemicellulose biosynthesis gene cluster	*-*	GALLO_0364	SGGB_0392	SGGBAA2069_c03530	-	SMA_0392 (p)	Sinf_0344
	*-*	GALLO_0365	SGGB_0393 (p)	SGGBAA2069_c03540 (s)	-	SMA_0393 (p)	-
			SGGB_0394 (p)	SGGBAA2069_c03550 (s)			
	*-*	GALLO_0366	SGGB_0395	SGGBAA2069_c03560	-	SMA_0394 (p)	Sinf_0345
							Sinf_0346 (s)
	*-*	GALLO_0367	SGGB_0396	SGGBAA2069_c03570	-	-	-
Hemolysin TLY	*-*	GALLO_0630	SGGB_0605	SGGBAA2069_c05730	SGPB_0499	SMA_0591	Sinf_0511
Hemolysin III	*-*	GALLO_1262	SGGB_1256	SGGBAA2069_c12530	SGPB_1172	SMA_1191	Sinf_1093
Hemolysin A family protein	*-*	GALLO_1799	SGGB_1786	SGGBAA2069_c17570	SGPB_1603	SMA_1706	Sinf_1530
Exfoliative toxin B	*-*	-	-	-	-	-	Sinf_0933
Macrophage infectivity potentiator protein	*-*	-	-	-	-	-	Sinf_0931

(a) Not found; (p) Pseudogenes; (s) Split CDSs corresponding to fragments of the original gene not yet characterized as pseudogenes; (t) Truncated.

More genes whose products may be implicated in other interactions with the host cells beyond adherence could be identified. Despite the fact that the SBSEC members are considered non-hemolytic (as members of the group D streptococci), *S. gallolyticus* ATCC BAA-2069 has been reported to cause alpha-hemolysis on Schaedler Agar with 5% sheep blood [54]. Three hemolysins are conserved among the SBSEC members (Table 5). Sequence analysis of Sinf_1513 and Sinf_1683, also annotated as hemolysin genes, was not supportive of a hemolysin protein product (data not shown). Apart from hemolysins, a putative exfoliative toxin B (Sinf_0933) and a macrophage infectivity potentiator protein (Sinf_0931) are present in the *S. infantarius* genome [20]. Similar genes can be found in *S. thermophilus* strains but not in the other SBSEC species and in our opinion functional analysis is required to verify these annotations.

In order to expand our investigation for putative pathogenicity traits, we screened the genomes of *S. macedonicus* and its related SBSEC species using the VFDB (virulence factors database) [55] and the genes determined to encode putative VFs during this analysis are presented in Additional file 11: Table S7. Current results of comparative pathogenomics have allowed the classification of available streptococcal VFs in nine categories, i.e. adhesion factors, DNases, exoenzymes, immune evasion factors, immunoreactive antigens, factors involved in metal transport, proteases, superantigens and toxins [56]. The general profile of VFs for the six streptococci under investigation was rather similar and we determined a number of previously unidentified potential VFs dispersed among all or some of the SBSEC members. Several of these genes coding for putative VFs like the agglutinin receptor, the fibronectin/fibrinogen-binding protein (*fbp54*/*pavA*), the lipoprotein rotamase A (*slrA*), the plasmin receptor/GAPDH multifunctional protein, the streptococcal enolase exoenzyme, the pneumococcal surface antigen A and specific proteases (i.e. *cppA*, *htrA*/*degP* and *tig*/*ropA*) have been experimentally correlated with the virulence of pathogenic streptococci beyond SBSEC members [57-67]. Some genes were also involved in the production of a capsule that enables bacterial cells to evade phagocytosis (Additional file 11: Table S7) [68]. According to our analysis, all SBSEC streptococci carry a main gene cluster spanning practically the same position in the chromosome that could be involved in the biosynthesis of a capsule (Additional file 12: Figure S5). Even though the *cps* clusters are identical between *S. gallolyticus* UCN34 and ATCC BAA-2069 [54], multiple sequence alignment indicates significant structural diversity in the rest of the strains. The existence of dispersed pseudogenes in the gene clusters of *S. infantarius* and *S. macedonicus* (e.g. SMA_0865 and SMA_0866) may prohibit the production of capsule substances. It should be emphasized that the strains of the SBSEC missed hits in several major categories of streptococcal VFs (e.g. DNases, immunoreactive antigens, superantigens and toxins) supporting a reduced pathogenic potential for the SBSEC in general.

## Conclusions

In this study we presented the analysis of the first complete genome sequence of a dairy isolate of *S. macedonicus*. While comparative analysis among specific subgroups of the SBSEC species has been previously presented [20,22,25,54], comparative genomics of the six complete genome sequences was missing. Most importantly, the inclusion of *S. macedonicus* into this analysis provided a better opportunity to assess niche adaptation of the SBSEC species that was so far limited by the presence of only one dairy isolate (i.e. *S. infantarius* CJ18) among four clinical strains.

Our findings clearly support two distinct evolutionary patterns within the SBSEC. On the one hand, *S. gallolyticus* is a species without apparent genome decay and the available genomes suggest that it is a robust bacterium able to thrive in the rumen of herbivores. On the other hand, the remaining SBSEC species, i.e. *S. macedonicus*, *S. pasteurianus* and *S. infantarius* exhibit decreased genome sizes accompanied by increased percentages of potential pseudogenes due to extensive genome decay, suggesting adaptation to nutrient-rich environments. This does not necessarily mean that the environment to which the three species have been adapted is the same. The three species appear with a reduced ability to catabolize complex plant carbohydrates and to detoxify substances met in the rumen, which indicates that they must have deviated from this niche. It has been proposed that *S. pasteurianus* may now reside in the human gut [22], while *S. infantarius* presents adaptations to milk [20]. *Streptococcus macedonicus* also possesses traits that may contribute to growth in the dairy environment, like the extra lactose gene cluster and its proteolytic system. However, all SBSEC strains, including clinical isolates, seem to be competent in the metabolism of lactose and galactose or the degradation of milk proteins. Taking into account these shared characteristics of all SBSEC species, it is tempting to speculate that their common ancestor may have been able to grow in milk.

In our opinion, several genome traits per se suggest adaptation of *S. macedonicus* to milk. This hypothesis is also supported by the predicted interspecies interactions of *S. macedonicus* with other bacteria. As it has been recently reported for *S. infantarius*[20], the *S. macedonicus* genome may have acquired genes originating from *L. lactis* and *S. thermophilus* through HGT. The predicted exposure of *S. macedonicus* to *S. thermophilus* phages, based on our CRISPR sequence analysis, is also in favor of this theory. No such evidence was found for the rest of the SBSEC members apart from *S. infantarius*. These findings are in accordance with the frequent isolation of *S. macedonicus* from dairy products [13] and the prevalence of *S. infantarius* in certain African fermented milks [20]. One additional question that arises is whether *S. macedonicus* and *S. infantarius* are specialized dairy microbes like *S. thermophilus*. We believe that the available data does not support this idea. Traits of milk adaptation have been shown to be strain-specific in *S. infantarius*[20]. In addition, the genome size of *S. macedonicus* is significantly larger, containing a higher number of functional genes in comparison to *S. thermophilus. Streptococcus macedonicus* and *S. infantarius* may thus represent intermediate evolutionary stages analogous to those followed by the ancestors of *S. thermophilus* before it became today’s starter culture.

Thus, the safety concerns raised from the presence of SBSEC members in foods remain, even if reports implicating *S. macedonicus* with disease are rather scarce [69,70]. Our comparative genomic analysis showed that both *S. macedonicus* and *S. infantarius* miss several VFs that are highly conserved in *S. gallolyticus*. However, the interpretation of these findings becomes complicated as the available genome of the human blood isolate *S. pasteurianus* ATCC 43144 also exhibited diminished traits of pathogenicity similarly to the two dairy SBSEC members. Overall, our analysis provides evidence in agreement with the clinical perception that the members of the SBSEC are lower grade streptococcal pathogens [10]. In terms of food safety, the dairy SBSEC could thus constitute a risk factor similar to the presence of enterococci that are widely found in fermented products, but cause no major problem for the average healthy and adult consumer. Nevertheless, it is the correlation of the SBSEC microorganisms with human endocarditis and colon cancer in particular that may require special considerations. For example, it has been proposed that members of the SBSEC like *S. gallolyticus* may be part of the etiology of colon cancer by causing chronic inflammation [10]. In order to assess the pathogenicity of this group of streptococci, more research is needed on the specific mechanisms employed by SBSEC members to cause disease. More comparative and functional genomics studies comprising SBSEC genomes are necessary that will cover additional species of the complex, like the recently sequenced *Streptococcus lutetiensis*[71]. New clinico-epidemiological studies should also be undertaken in view of the most recent changes in the taxonomy of the SBSEC complex [72]. In the meantime, assuming the worse case scenario, we propose that the presence of SBSEC members including *S. macedonicus* and *S. infantarius* in foods should be avoided until their pathogenicity status is resolved.

## Methods

### Sequencing and annotation of the genome of *Streptococcus macedonicus* ACA-DC 198

The genome of *S. macedonicus* ACA-DC 198 was sequenced and annotated as described previously [19]. In brief, we employed a sequencing strategy involving shotgun/paired-end pyrosequencing and shotgun Illumina sequencing with the 454 GS-FLX (Roche Diagnostics, Basel, Switzerland) and the Hiseq 2000 (Illumina, San Diego, CA), respectively. Sequences were assembled in two contigs corresponding to the complete genome sequence and the pSMA198 plasmid of *S. macedonicus*. The hybrid assembly was validated against an *Nhe*I optical map of the *S. macedonicus* genome generated at OpGen Technologies, Inc. (Madison, WI). The genome was annotated using the RAST [73] and the Basys [74] pipelines. Predictions of the two pipelines were compiled into a single annotation file after manual curation in the Kodon software environment (Applied Maths N.V., Sint-Martens-Latem, Belgium). Final corrections and quality assessment of the annotation were performed with the GenePRIMP pipeline [21]. GenePRIMP was also used for the identification of putative pseudogenes. The circular map of the *S. macedonicus* genome was generated by the DNAPlotter software [75].

### Comparative genomics of *Streptococcus macedonicus* ACA-DC 198 against related members of the SBSEC

The complete genome sequence of *S. macedonicus* was compared to those of *S. gallolyticus* strains UCN34, ATCC 43143 and ATCC BAA-2069, *S. pasteurianus* ATCC 43144 and *S. infantarius* CJ18 using a variety of tools. In order to visualize conserved genomic regions or chromosomal rearrangements, whole genome sequence alignments were performed by progressiveMAUVE [24]. Estimation of the differential gene content of the genomes, as well as whole genome phylogeny of streptococci was carried out within the EDGAR software framework [23]. Venn diagrams were designed with the VennDiagram package in R [76]. The glycobiome of the SBSEC members was determined based on the pre-computed data available in the CAZy database [26].

### Additional analysis

Sequence similarity searches were performed with the BLAST suite [77]. Whenever necessary, protein sequences were analyzed in the CDD [78]. Figures showing similarity of gene clusters were constructed with the Easyfig comparison visualizer [79]. Potential VFs included in the VFDB [55] were identified in the SBSEC genomes with mpiBLAST, as implemented in the mGenomeSubtractor website [80]. In brief, the entire VFDB was uploaded as the reference sequence in the mGenomeSubtractor website and each genome was used as the query sequence. Only hits with *H*-value homology score > 0.6 were considered significant. CRISPRs were analyzed by the tools available in the CRISPRcompar web-service [81]. A general bit score cutoff value of 42.0 was applied during BLASTN of CRISPR spacers. GIs were identified and visualized by the IslandViewer application that utilizes three different prediction tools (i.e. IslandPick, SIGI-HMM and IslandPath-DIMOB) relying on either sequence composition or comparative genomics [36]. Genomic regions of RM systems were determined in the REBASE genomes database [82].

### Availability of supporting data

The data set supporting the phylogenetic tree presented in Additional file 1: Figure S1 of this article is available in the [Dryad] repository, [unique persistent identifier doi:10.5061/dryad.7d039 and hyperlink to datasets in http://datadryad.org/]. Additional data sets supporting the results of this article are included within the article and its additional files.

## Abbreviations

SBSEC: *Streptococcus bovis*/*Streptococcus equinus* complex; LAB: Lactic acid bacteria; GRAS: Generally regarded as safe; QPS: Qualified presumption of safety; GIT: Gastrointestinal tract; GAS: Group A streptococci; GBS: Group B streptococci; CDS: Coding DNA sequence; LCB: Local collinear block; HGT: Horizontal gene transfer; PEP-PTS: Phosphoenolpyruvate-dependent phosphotransferase system; CEP: Cell-envelope associated proteinase; PMF: Proton motive force; CRISPR: Clustered regularly interspaced short palindromic repeats; GI: Genomic island; RM: Restriction modification; VF: Virulence factor; ECM: Extracellular matrix; MSCRAMM: Microbial surface recognizing adhesive matrix molecule; CDD: Conserved domain database; VFDB: Virulence factors database.

## Competing interests

SF is an employee of Genoscreen and PS is a consultant of the same company. BP is also an employee of Applied Maths NV.

## Authors’ contributions

KP initiated the project, performed the genome analysis and comparative genomics and wrote the manuscript; RA isolated the genomic DNA and critically revised the manuscript; EM assisted in the genome analysis and helped to draft the manuscript; JB performed analysis with the EDGAR software; NCP helped with data interpretation and critically revised the manuscript; SJH helped with data interpretation and critically revised the manuscript; SF performed the sequencing of the genome and participated in the genome assembly; PR helped with data interpretation and critically revised the manuscript; PS helped with data interpretation and critically revised the manuscript; BP initiated the project, helped with data interpretation and critically revised the manuscript; ET initiated the project, helped with data interpretation and critically revised the manuscript. All authors have read and approved the final manuscript.

## Supplementary Material

Additional file 1: Figure S1 Whole genome phylogeny of the *Streptococcus* genus. The phylogenetic tree was constructed using the EDGAR tool based on complete genome sequences of streptococci. The branch of the members of the *Streptococcus bovis*/*Streptococcus equinus* complex (SBSEC) is delimited by a bracket. Click here for file

Additional file 2: Table S1 Core genome analysis among *Streptococcus gallolyticus* UCN34, *Streptococcus gallolyticus* ATCC 43143, *Streptococcus gallolyticus* ATCC BAA-2069, *Streptococcus macedonicus* ACA-DC 198 and *Streptococcus pasteurianus* ATCC 43144 calculated using the EDGAR software. Click here for file

Additional file 3: Table S2 Core genome analysis among *Streptococcus gallolyticus* ATCC 43143, *Streptococcus infantarius* CJ18, *Streptococcus macedonicus* ACA-DC 198 and *Streptococcus pasteurianus* ATCC 43144 calculated using the EDGAR software. In this analysis *S. gallolyticus* ATCC 43143 was selected as a representative of the *S. gallolyticus* species, since it has the longest genome size among the three sequenced strains. Click here for file

Additional file 4: Table S3 Glycobiome analysis of *Streptococcus gallolyticus* UCN34, *Streptococcus gallolyticus* ATCC 43143, *Streptococcus gallolyticus* ATCC BAA-2069, *Streptococcus pasteurianus* ATCC 43144, *Streptococcus macedonicus* ACA-DC 198 and *Streptococcus infantarius* CJ18 using the CAZy database. Click here for file

Additional file 5: Figure S2 Circular maps of the *Streptococcus bovis*/*Streptococcus equinus* complex genomes highlighting the regions corresponding to genomic islands (GIs). GIs are coloured within the circular maps according to the tool that predicted each one of them: green, orange and blue were predicted with IslandPick, SIGI-HMM and IslandPath-DIMOB, respectively. The integrated GIs are presented at the periphery of the map in red colour. The black line plot represents the GC content (%) of the genomic sequences. Numbering of the GIs for each genome starts from the first GI found after position 0 of the genome in a clockwise direction. Click here for file

Additional file 6: Figure S3 Analysis of the genomic island (GI) 4 of *Streptococcus macedonicus* ACA-DC 198 presented as an example of a GI potentially originating from multiple donors. In the graphical summary of the BLASTN results arrows indicate the best BLASTN hits with > 90% sequence identity corresponding to: a. *Streptococcus thermophilus* MN-ZLW-002 genomic sequence (96% sequence identity); b. *Lactococcus garvieae* 21881 plasmid pGL3 sequence (98% sequence identity); c. *Streptococcus intermedius* B196 genomic sequence (96% sequence identity); d. *Streptococcus thermophilus* MN-ZLW-002 genomic sequence (99% sequence identity) and e. *Streptococcus thermophilus* MN-ZLW-002 genomic sequence (99% sequence identity). Click here for file

Additional file 7: Table S4 Genes within each integrated GI of *Streptococcus macedonicus* ACA-DC 198 as determined by IslandViewer. Click here for file

Additional file 8: Table S5 The singleton genes of *Streptococcus macedonicus* ACA-DC 198 calculated against the other members of the *Streptococcus bovis*/*Streptococcus equinus* complex (SBSEC) using the EDGAR software. The singleton genes of *S. macedonicus* were calculated twice, taking or not into account the genome of *S. infantarius*. Thus, genes shared only by *S. macedonicus* and *S. infantarius* among the SBSEC members also appear in the table. Click here for file

Additional file 9: Figure S4 Circular maps of the *Streptococcus bovis*/*Streptococcus equinus* complex genomes highlighting the regions corresponding to restriction modification systems (RMs). RMs are presented as predicted in the REBASE database. Colours and symbols are exemplified at the bottom of the figure. Click here for file

Additional file 10: Table S6 Comparison of the CRISPR/Cas systems among members of the *Streptococcus bovis*/*Streptococcus equinus* complex using CRISPRcompar. Click here for file

Additional file 11: Table S7 Genes in the *Streptococcus bovis*/*Streptococcus equinus* complex identified as virulence factors within the VFDB. Click here for file

Additional file 12: Figure S5 Multiple sequence alignment of the capsule biosynthetic gene cluster found in the genomes of the *Streptococcus bovis*/*Streptococcus equinus* complex after BLASTN analysis. Grey shading represents the % identity among the nucleotide sequences according to the colour gradient presented at the lower right corner of the figure. Potential pseudogenes are marked with a "p". Click here for file
