# Supplementary figures and images for: Comparative genomics of the dairy isolate Streptococcus macedonicus ACA-DC 198 against related members of the Streptococcus bovis/Streptococcus equinus complex
Source: BMC Genomics. 2014 Apr 8;15:272. doi: 10.1186/1471-2164-15-272 (PMC4051162; doi:10.1186/1471-2164-15-272)

Figure S1

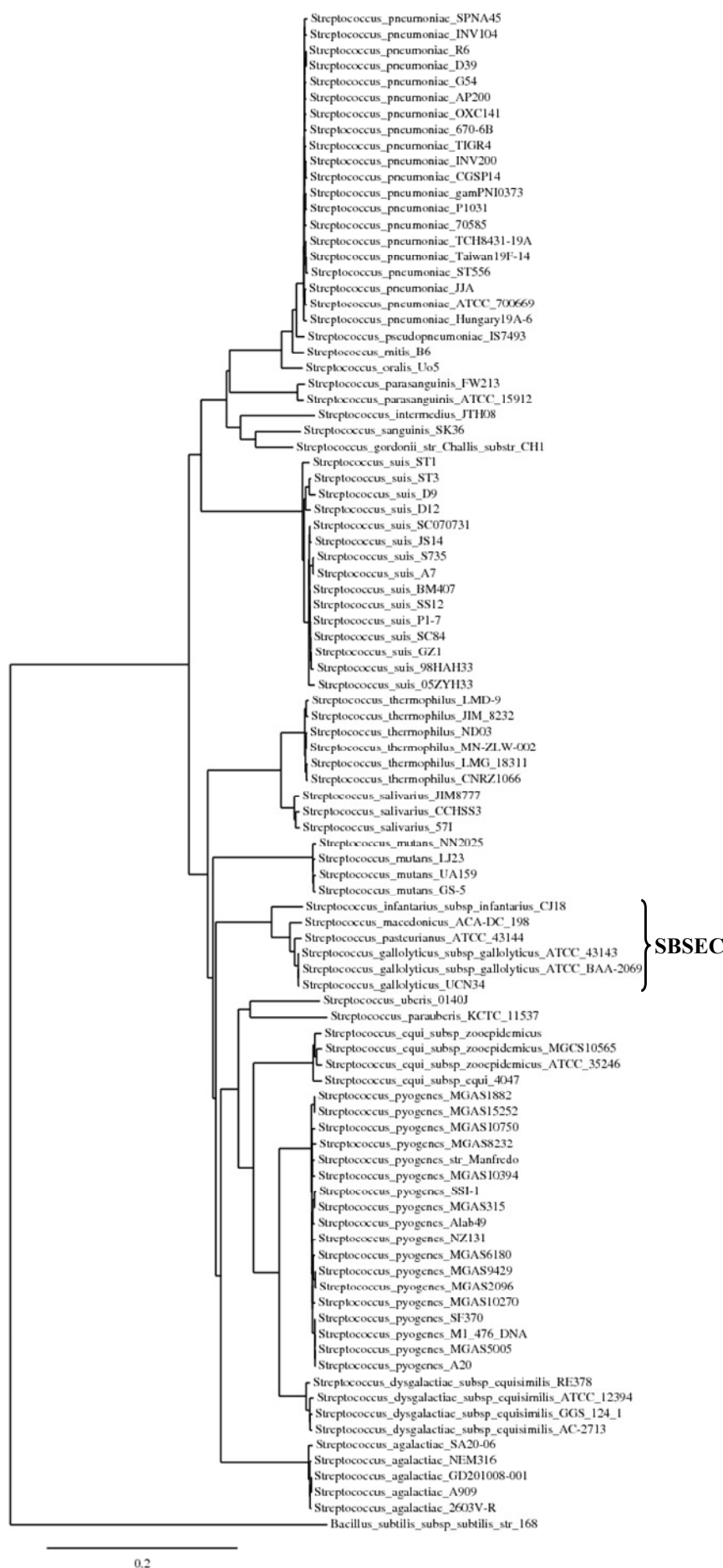

Supplement: Additional file 1: Figure S1 — Whole genome phylogeny of the Streptococcus genus. The phylogenetic tree was constructed using the EDGAR tool based on complete genome sequences of streptococci. The branch of the members of the Streptococcus bovis/Streptococcus equinus complex (SBSEC) is delimited by a bracket. [file 1471-2164-15-272-S1.PDF]

Figure S2

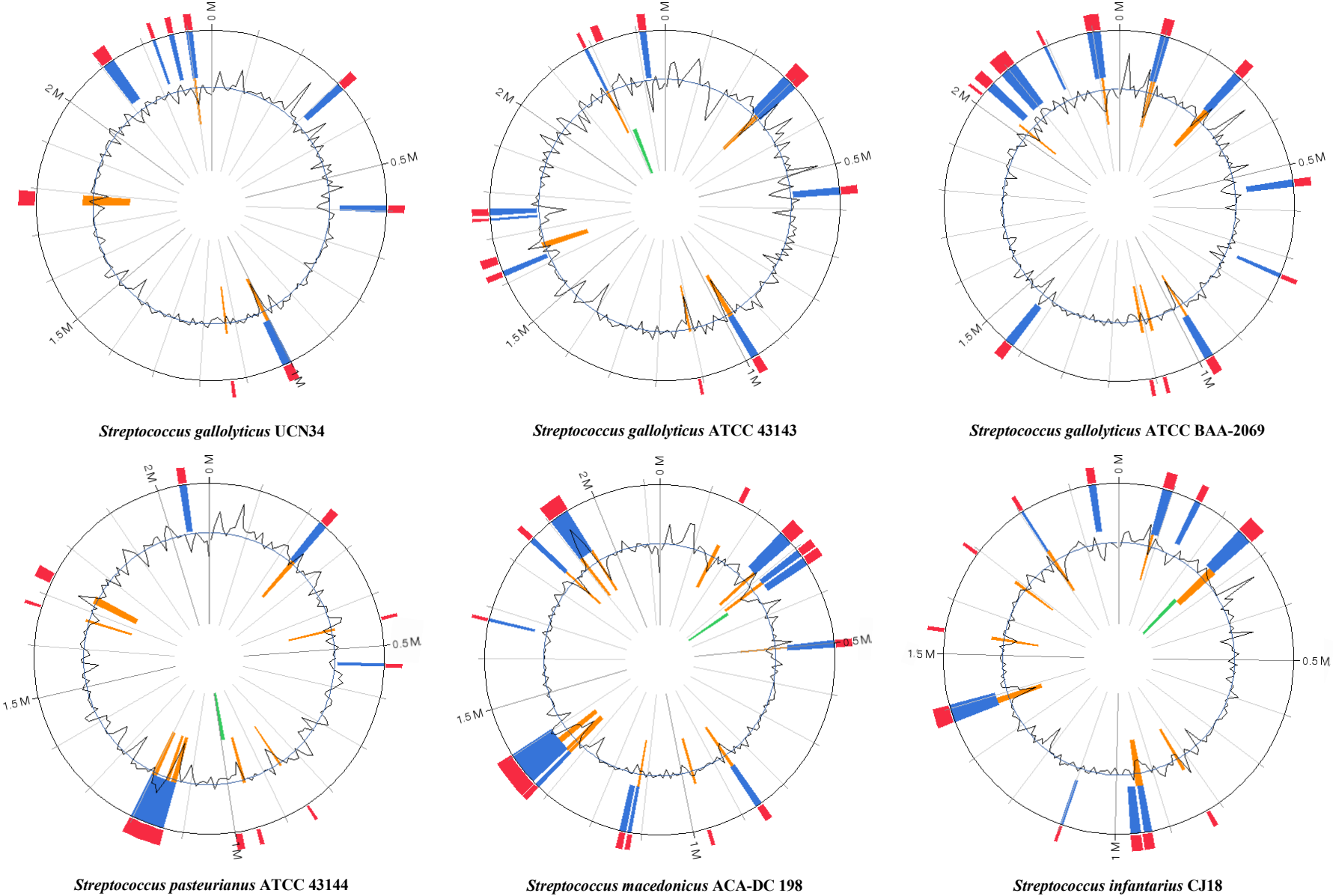

Supplement: Additional file 5: Figure S2 — Circular maps of the Streptococcus bovis/Streptococcus equinus complex genomes highlighting the regions corresponding to genomic islands (GIs). GIs are coloured within the circular maps according to the tool that predicted each one of them: green, orange and blue were predicted with IslandPick, SIGI-HMM and IslandPath-DIMOB, respectively. The integrated GIs are presented at the periphery of the map in red colour. The black line plot represents the GC content (%) of the genomic sequences. Numbering of the GIs for each genome starts from the first GI found after position 0 of the genome in a clockwise direction. [file 1471-2164-15-272-S5.PDF]

Figure S3

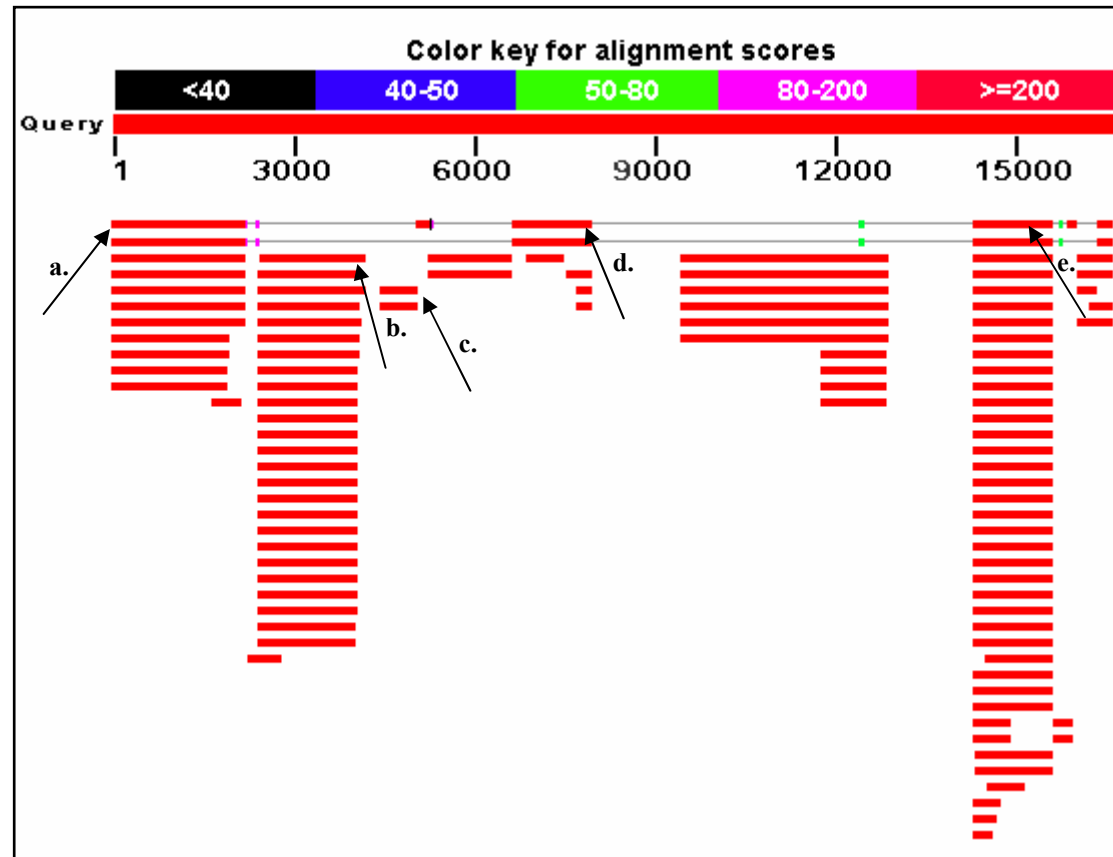

Supplement: Additional file 6: Figure S3 — Analysis of the genomic island (GI) 4 of Streptococcus macedonicus ACA-DC 198 presented as an example of a GI potentially originating from multiple donors. In the graphical summary of the BLASTN results arrows indicate the best BLASTN hits with > 90% sequence identity corresponding to: a. Streptococcus thermophilus MN-ZLW-002 genomic sequence (96% sequence identity); b. Lactococcus garvieae 21881 plasmid pGL3 sequence (98% sequence identity); c. Streptococcus intermedius B196 genomic sequence (96% sequence identity); d. Streptococcus thermophilus MN-ZLW-002 genomic sequence (99% sequence identity) and e. Streptococcus thermophilus MN-ZLW-002 genomic sequence (99% sequence identity). [file 1471-2164-15-272-S6.PDF]

Figure S4

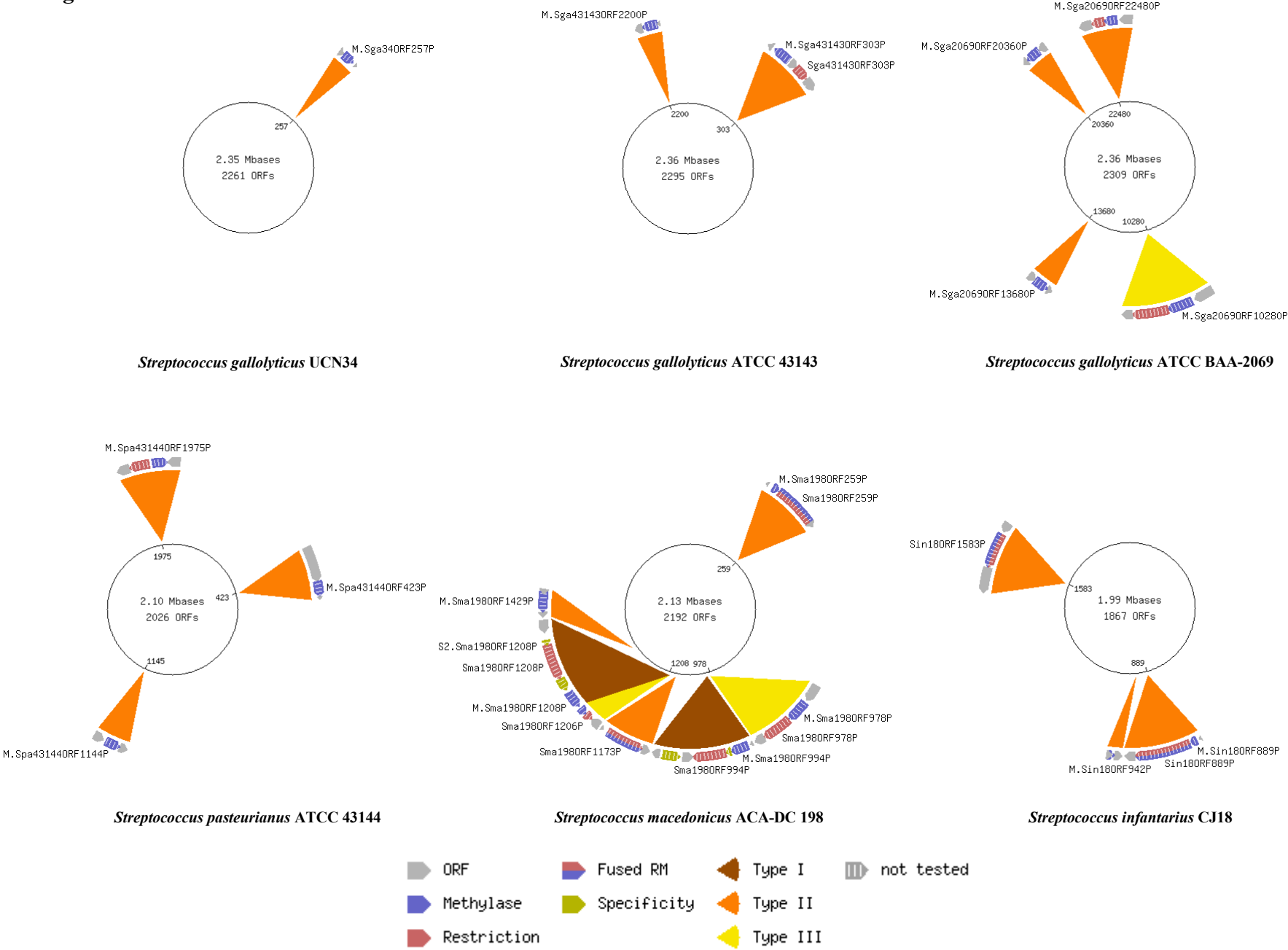

Supplement: Additional file 9: Figure S4 — Circular maps of the Streptococcus bovis/Streptococcus equinus complex genomes highlighting the regions corresponding to restriction modification systems (RMs). RMs are presented as predicted in the REBASE database. Colours and symbols are exemplified at the bottom of the figure. [file 1471-2164-15-272-S9.PDF]

Figure S5

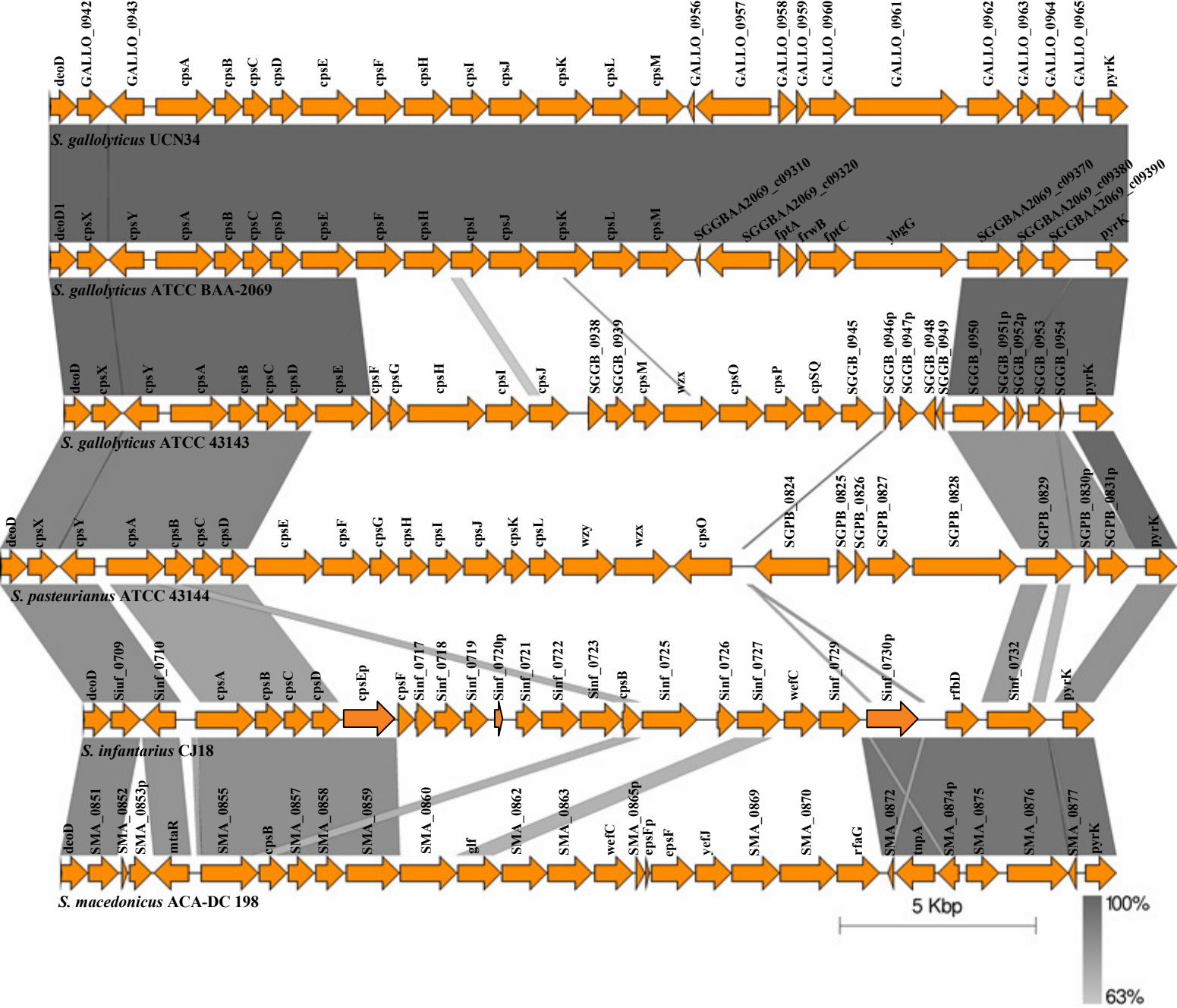

Supplement: Additional file 12: Figure S5 — Multiple sequence alignment of the capsule biosynthetic gene cluster found in the genomes of the Streptococcus bovis/Streptococcus equinus complex after BLASTN analysis. Grey shading represents the % identity among the nucleotide sequences according to the colour gradient presented at the lower right corner of the figure. Potential pseudogenes are marked with a "p". [file 1471-2164-15-272-S12.PDF]
