# Supplementary material for: Comparative genomics of the dairy isolate Streptococcus macedonicus ACA-DC 198 against related members of the Streptococcus bovis/Streptococcus equinus complex
Source: BMC Genomics. 2014 Apr 8;15:272. doi: 10.1186/1471-2164-15-272 (PMC4051162; doi:10.1186/1471-2164-15-272)
Supplement: Additional file 10: Table S6 — Comparison of the CRISPR/Cas systems among members of the Streptococcus bovis/Streptococcus equinus complex using CRISPRcompar. [file 1471-2164-15-272-S10.DOC]

**Table S6. Comparison of the CRISPR/Cas systems among members of the *Streptococcus bovis*/*Streptococcus equinus* complex using CRISPRcompar**

|  | ***Streptococcus gallolyticus* UCN34** | | ***Streptococcus gallolyticus* subsp. *gallolyticus***  **ATCC 43143** | | ***Streptococcus gallolyticus* subsp. *gallolyticus***  **ATCC BAA-2069** | | ***Streptococcus pasteurianus***  **ATCC 43144** | | ***Streptococcus macedonicus***  **ACA-DC 198** | | ***Streptococcus infantarius* subsp. *infantarius* CJ18** | |
| --- | --- | --- | --- | --- | --- | --- | --- | --- | --- | --- | --- | --- |
| **CRISPR**  **label** | **Position^a^** | **Nbr Spacers^b^** | **Position** | **Nbr Spacers** | **Position** | **Nbr Spacers** | **Position** | **Nbr Spacers** | **Position** | **Nbr Spacers** | **Position** | **Nbr Spacers** |
| **CRISPR_1** | 1507890,  1508913 | 15 |  |  |  |  |  |  | 1412482,  1415817 | 50 |  |  |
| **CRISPR_2** | 1515490,  1516317 | 12 | 1484496,  1486444 | 29 | 1517213,  1518237 | 15 |  |  |  |  |  |  |
| **CRISPR_3** |  |  | 1477224,  1477919 | 10 |  |  |  |  |  |  |  |  |
| **CRISPR_4** |  |  |  |  | 1515726,  1516570 | 12 |  |  |  |  |  |  |
| **CRISPR_5** |  |  |  |  |  |  |  |  |  |  | 1273106,  1273801 | 10 |
| **CRISPR_6** |  |  |  |  |  |  | 1395041,  1397515 | 37 |  |  |  |  |

^a^Genomic coordinates of the CRISPR locus

^b^Number of spacers
