# Supplementary material for: Comparative genomics of the dairy isolate Streptococcus macedonicus ACA-DC 198 against related members of the Streptococcus bovis/Streptococcus equinus complex
Source: BMC Genomics. 2014 Apr 8;15:272. doi: 10.1186/1471-2164-15-272 (PMC4051162; doi:10.1186/1471-2164-15-272)
Supplement: Additional file 11: Table S7 — Genes in the Streptococcus bovis/Streptococcus equinus complex identified as virulence factors within the VFDB. [file 1471-2164-15-272-S11.DOC]

**Table S7. Genes in the *Streptococcus bovis*/*Streptococcus equinus* complex identified as virulence factors within the VFDB**

| **Virulence factor category/**  **Virulence factors** | **Gene** | ***S. gallolyticus* UCN34** | ***S*. *gallolyticus* ATCC 43143** | ***S*. *gallolyticus* ATCC BAA-2069** | ***S. pasteurianus* ATCC 43144** | ***S. macedonicus* ACA-DC 198** | ***S*. *infantarius* CJ18** |
| --- | --- | --- | --- | --- | --- | --- | --- |
| **Adhesins/** |  |  |  |  |  |  |  |
| **agglutinin receptor** | - (a) | - | - | - | SGPB_1131 | SMA_1415 | - |
| **fibronectin-binding proteins** | *fbp54/ pavA* | GALLO_0841 | SGGB_0828 | SGGBAA2069_c08170 | SGPB_0720 | SMA_0775 | Sinf_0656 |
| **rlrA islet** | *srtC* | GALLO_2177 | SGGB_2209 | SGGBAA2069_c21740 | - | - | - |
| **sortase A** | *srtA* | GALLO_0299 GALLO_1127 | SGGB_1117 SGGB_2153 | SGGBAA2069_c11150 SGGBAA2069_c20330 | SGPB_0986 | SMA_1044 | Sinf_0968 |
| **streptococcal glucosyltransferases** | *gtfD* | GALLO_1057 | SGGB_1046 | SGGBAA2069_c10420 | - | - | Sinf_0877 |
| **streptococcal lipoprotein rotamase A** | *slrA* | GALLO_1717 | SGGB_1704 | SGGBAA2069_c16750 | SGPB_1517 | SMA_1626 | Sinf_1457 |
| **streptococcal plasmin receptor/GAPDH** | *plr*/*gapA* | GALLO_1996 | SGGB_1980 | - | - | SMA_1891 | - |
|  |  |  |  |  |  |  |  |
| **Exoenzymes/** |  |  |  |  |  |  |  |
| **streptococcal enolase** | *eno* | GALLO_1458 | SGGB_1454 | - | - | SMA_1460 | - |
|  |  |  |  |  |  |  |  |
| **Immune evasion/** |  |  |  |  |  |  |  |
| **capsule** | - | GALLO_0317 GALLO_0829 GALLO_0830 GALLO_0831 GALLO_0943 GALLO_0944 GALLO_0945 GALLO_0946 GALLO_0947 GALLO_0948 GALLO_0949 GALLO_1375 GALLO_1377 GALLO_1378 GALLO_1379 GALLO_1380 GALLO_1381 | SGGB_0345 SGGB_0815 SGGB_0817 SGGB_0927 SGGB_0928 SGGB_0929 SGGB_0930 SGGB_0931 SGGB_0932 SGGB_0933 SGGB_0934 SGGB_1369 SGGB_1371 SGGB_1372 SGGB_1373 SGGB_1374 SGGB_1375 SGGB_2077 | SGGBAA2069_c03050 SGGBAA2069_c08040 SGGBAA2069_c08050 SGGBAA2069_c09180 SGGBAA2069_c09190 SGGBAA2069_c09200 SGGBAA2069_c09210 SGGBAA2069_c09220 SGGBAA2069_c09230 SGGBAA2069_c09240 SGGBAA2069_c14000 SGGBAA2069_c14020 SGGBAA2069_c14030 SGGBAA2069_c14040 SGGBAA2069_c14050 SGGBAA2069_c14060 SGGBAA2069_c20890 | SGPB_0269 SGPB_0693 SGPB_0694 SGPB_0808 SGPB_0809 SGPB_0810 SGPB_0811 SGPB_0812 SGPB_0813 SGPB_0823 SGPB_1295 SGPB_1296 SGPB_1297 SGPB_1298 SGPB_1299 SGPB_1300 SGPB_1301 SGPB_1885 | SMA_0346 SMA_0762 SMA_0763 SMA_0764 SMA_0854 SMA_0855 SMA_0856 SMA_0857 SMA_0858 SMA_0859 SMA_0861 SMA_0862 SMA_0863 SMA_0875 SMA_1308 SMA_1311 SMA_1314 SMA_1315 SMA_1316 SMA_1317 SMA_1318 SMA_2054 | Sinf_0298 Sinf_0647 Sinf_0648 Sinf_0710 Sinf_0711 Sinf_0712 Sinf_0713 Sinf_0714 Sinf_0716 Sinf_0717 Sinf_0718 Sinf_0727 Sinf_0731 Sinf_1192 Sinf_1197 Sinf_1198 Sinf_1835 |
|  |  |  |  |  |  |  |  |
| **Metal transport/** |  |  |  |  |  |  |  |
| **pneumococcal surface antigen A- metal binding protein SloC** | *psaA* | GALLO_2047 | SGGB_2030 | SGGBAA2069_c20050 | SGPB_1854 | - | - |
|  |  |  |  |  |  |  |  |
| **Protease/** |  |  |  |  |  |  |  |
| **C3-degrading protease** | *cppA* | GALLO_1957 | SGGB_1940 | SGGBAA2069_c19070 | SGPB_1789 | SMA_1858 | Sinf_1674 |
| **serine protease** | *htrA*/*degP* | GALLO_2261 | SGGB_2294 | SGGBAA2069_c23080 | SGPB_2025 | SMA_2191 | Sinf_1963 |
| **trigger factor** | *tig*/*ropA* | GALLO_0251 | SGGB_0324 | SGGBAA2069_c02840 | SGPB_0244 | SMA_0290 | Sinf_0279 |

(a) Not found
